# Establishment and genomic characterization of a sporadic malignant peripheral nerve sheath tumor cell line

**DOI:** 10.1038/s41598-021-85055-2

**Published:** 2021-03-11

**Authors:** Jody Fromm Longo, Stephanie N. Brosius, Iya Znoyko, Victoria A. Alers, Dorea P. Jenkins, Robert C. Wilson, Andrew J. Carroll, Daynna J. Wolff, Kevin A. Roth, Steven L. Carroll

**Affiliations:** 1 Department of Pathology and Laboratory Medicine, Medical University of South Carolina, 171 Ashley Avenue, MSC 908, Charleston, SC 29425-9080 USA; 2 Center for Genomic Medicine, Medical University of South Carolina, Charleston, SC 29425-9080 USA; 3 Department of Pathology, University of Alabama at Birmingham, Birmingham, AL 35294-0017 USA; 4 Department of Genetics, University of Alabama at Birmingham, Birmingham, AL 35294-0017 USA; 5 Medical Scientist Training Program, University of Alabama at Birmingham, Birmingham, AL 35294-0017 USA; 6 Department of Pathology and Cell Biology, Vagelos College of Physicians and Surgeons, Columbia University, New York, NY 10032 USA; 7 Present Address: Division of Neurology, Children’s Hospital of Philadelphia, Philadelphia, PA 19104 USA

**Keywords:** Cancer genomics, Cancer models, Sarcoma

## Abstract

Malignant peripheral nerve sheath tumors (MPNSTs) are aggressive Schwann cell-derived neoplasms that occur sporadically or in patients with neurofibromatosis type 1 (NF1). Preclinical research on sporadic MPNSTs has been limited as few cell lines exist. We generated and characterized a new sporadic MPNST cell line, 2XSB, which shares the molecular and genomic features of the parent tumor. These cells have a highly complex karyotype with extensive chromothripsis. 2XSB cells show robust invasive 3-dimensional and clonogenic culture capability and form solid tumors when xenografted into immunodeficient mice. High-density single nucleotide polymorphism array and whole exome sequencing analyses indicate that, unlike NF1-associated MPNSTs, 2XSB cells have intact, functional *NF1* alleles with no evidence of mutations in genes encoding components of Polycomb Repressor Complex 2. However, mutations in other genes implicated in MPNST pathogenesis were identified in 2XSB cells including homozygous deletion of *CDKN2A* and mutations in *TP53* and *PTEN.* We also identified mutations in genes not previously associated with MPNSTs but associated with the pathogenesis of other human cancers. These include *DNMT1, NUMA1, NTRK1, PDE11A, CSMD3, LRP5* and *ACTL9*. This sporadic MPNST-derived cell line provides a useful tool for investigating the biology and potential treatment regimens for sporadic MPNSTs.

## Introduction

Malignant peripheral nerve sheath tumors (MPNSTs) are aggressive neoplasms derived from the Schwann cell lineage^1,2^. These malignancies represent approximately 2–5% of all soft tissue sarcomas^3,4^ and are encountered in three different clinical settings. About half (40–50%) of MPNSTs occur in patients with the autosomal dominant tumor susceptibility syndrome neurofibromatosis type 1 (NF1)^1^. MPNSTs are the most common malignancy encountered in adult NF1 patients—these individuals’ lifetime risk of developing an MPNST has been estimated at between 5 and 13%^2,3^. Another 40–47% of MPNSTs are sporadic, with the remaining 10–13% occurring at sites of previous radiation therapy^5–8^. Although there is an ongoing controversy as to whether patients with NF1-associated, sporadic or radiation-induced MPNSTs have a worse prognosis^4–7,9^, there is general agreement that MPNSTs have a poor outcome irrespective of the clinical setting, with multiple medical centers reporting that patients with these tumors have five year disease-free survival rates of between 34 and 60%^6,8,10–15^.

The histology of MPNSTs arising in these three different clinical settings is identical. However, there is evidence suggesting that NF1-associated and sporadic MPNSTs arise via distinct pathogenic mechanisms. At present, the clinical history and pathogenesis of NF1-associated MPNSTs is best understood. NF1 patients, who carry a deleterious mutation in one allele of the gene encoding the *NF1* tumor suppressor, develop benign plexiform neurofibromas when a “second hit” inactivates the remaining functional *NF1* gene in a cell within the Schwann cell lineage. This “second hit” mutation promotes Schwann cell proliferation and triggers physiologic changes in the *NF1*-null Schwann cells such as enhanced secretion of Kit ligand^16^. Kit ligand secretion promotes the recruitment of other cell types such as mast cells (the target of Kit ligand) into the nascent neurofibroma. Plexiform neurofibromas subsequently transform into MPNSTs when mutations occur in additional tumor suppressor genes encoding proteins that regulate the cell cycle (e.g., *CDKN2A*, *TP53*) and key cytoplasmic signaling cascades (e.g., *PTEN*, a key regulator of the PI3-kinase/Akt signaling pathway)^17^. Mutations of genes encoding components of Polycomb Repressor Complex 2 (PRC2) also occur in a major fraction of NF1-associated MPNSTs^18,19^, resulting in epigenetic alterations. Less is known about the molecular abnormalities that drive the pathogenesis of sporadic MPNSTs. In part, this is because comprehensive genomic analyses have been performed on only a very small number of these neoplasms. Those studies that are available indicate that while some sporadic MPNSTs have *NF1* loss and mutations of genes encoding PRC2 components^18,19^, these mutations are not uniformly present in sporadic MPNSTs. The natural history of sporadic MPNSTs is also distinct—sporadic MPNSTs typically arise de novo rather than from a pre-existing plexiform neurofibroma, and they occur in patients 25–30 years older than patients with NF1-associated MPNSTs. Defining the genomic abnormalities in sporadic MPNSTs is essential if we are to understand the pathogenesis of these tumors and develop new therapies that are effective against them. Ideally, some of this information would be obtained from well characterized cell lines derived from sporadic MPNSTs. The availability of these lines would support a variety of experimental approaches including the manipulation of candidate genes of interest so that their role in the biology of sporadic MPNSTs can be ascertained.

Although several cell lines derived from NF1-associated MPNSTs are available, very few sporadic MPNST cell lines have been generated and none of the sporadic MPNST cell lines that are available have undergone a characterization of their functional characteristics and genomic abnormalities. Since the advancement of our understanding of MPNST pathogenesis and biology is dependent on well characterized reagents, a characterization of the currently available sporadic MPNST cell lines must be performed. This characterization is also essential as it is increasingly acknowledged that misidentification of cell lines is a major source of inaccuracies in the scientific literature^20^. To address this need, here we describe the generation and characterization of a new sporadic MPNST cell line, including comprehensive genomic analyses of the mutations present in this new line and its parent tumor.

## Results

### Establishment and initial characterization of a new sporadic MPNST cell line

**Figure 1:**
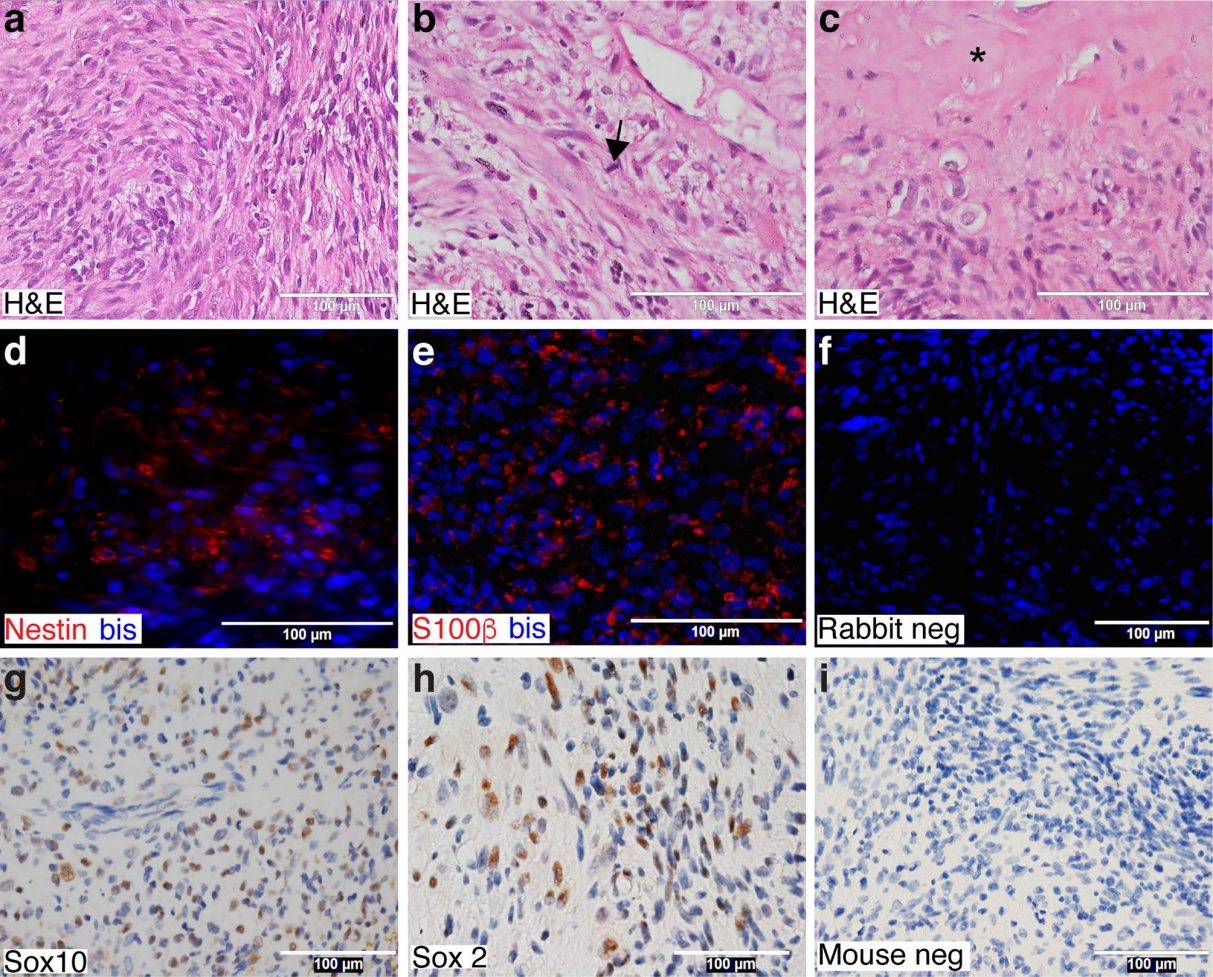
Pathology of the sporadic MPNST from which the 2XSB cell line was derived. (**a**) A representative hematoxylin and eosin (H&E) stained image (×40) displaying the densely packed, poorly differentiated cells composing this tumor. (**b**,**c**) High power (×60) H&E stained images highlighting high grade pathologic features such as mitotic figures (arrows, **b**) and necrosis (asterisk, **c**). (**d–f**) Representative immunohistochemical staining images of Schwann cell markers in formalin-fixed paraffin-embedded (FFPE) tumor tissue. Immunofluorescence staining highlights immunoreactivity for the intermediate filament nestin (**d**, red) and the calcium binding protein S100β (**e**, red), while immunoperoxidase staining shows nuclear immunoreactivity for the transcription factors Sox10 (**g**, brown) and Sox2 (**h**, brown). Non-immune rabbit (for S100β, Sox2) and mouse (for Sox10, nestin) IgGs were used as negative control primary antibodies (**f** and **i**, respectively). Bisbenzimide (“Bis”, blue) was used as a nuclear counterstain in (**d**)–(**f**). Hematoxylin was used as a nuclear counterstain in (**g**) to (**i**).

The cell line described below was established from a neoplasm (Fig. 1a) that arose in the right brachial nerve of a 57-year-old Caucasian woman with no previous history of cancer. The cells in this tumor were predominantly spindled, but some cells with a more polygonal morphology (left side, Fig. 1a) and occasional multinucleated giant cells were also present. The nuclei of the tumor cells were enlarged, hyperchromatic and pleomorphic (Fig. 1a,b). Brisk mitotic activity (> 4 mitoses per 10 high power fields) was readily identified (Fig. 1b, arrow) and focal areas of tumor necrosis were present (Fig. 1c, asterisk). To establish the diagnosis of this tumor, a broad differential was considered that included adult-type fibrosarcoma, leiomyosarcoma, epitheloid sarcoma, monophasic synovial sarcoma, melanoma and MPNST. Initial diagnostic immunostains of the tumor showed vimentin immunoreactivity and patchy staining for S100β. The tumor lacked the herringbone pattern characteristic of an adult-type fibrosarcoma; the presence of S100β immunoreactivity was also inconsistent with this diagnosis. The nuclei of the tumor cells did not have the blunt-ended morphology characteristic of a leiomyosarcoma. Further, although occasional leiomyosarcomas are immunoreactive for S100β, this tumor was negative for desmin, which ruled out this diagnostic possibility. The tumor also lacked cytokeratin immunoreactivity. This, together with the fact that the patient was not in the demographic where epitheloid sarcomas are most commonly encountered (adolescents and young adults 10–35 years of age), ruled out this possible diagnosis. To assess the possibility that this neoplasm might be a monophasic synovial sarcoma, we sequenced the transcriptome of the tumor and examined it for fusion genes using multiple algorithms (deFuse^21^, Tophat-Fusion^8^ and STAR-Fusion^22^). None of these analyses identified a SS18-SSX fusion gene, ruling out the possibility that this tumor was a monophasic synovial sarcoma.

The initial diagnostic workup showed that the tumor was not immunoreactive for the melanoma marker MART-1 (melan-A; MLANA). To further assess the possibility that this tumor was a melanoma, we examined RNA-Seq datasets from the parent tumor, the cell line established from the tumor (see below) and normal human Schwann cells for the expression of transcripts encoding MLANA and PMEL (premelanosome protein, the target of the HMB45 antibody). We compared these results to those from RNA-Seq datasets deposited in the COSMIC, NCBI, cBioPortal, the University of California Santa Cruz Genome Browser and NCBI that were derived from normal skin or melanomas. There was no evidence of *MLANA* or *PMEL* expression in the parent tumor, the cell line derived from the tumor or normal human Schwann cells [defined by RPKM (reads per kilobase of transcript per million reads mapped) values of less than 0.3, which is well below the cut-off for an expressed gene and 15- to 100-fold lower than what we found in normal skin or melanomas, respectively (Supplementary Fig. S1a)]. Consistent with this, transmission electron microscopy of cells derived from this tumor (see below) showed no evidence of melanosomes or premelanosomes. These findings indicated that the tumor was not a melanoma.

Having excluded the diagnostic possibilities noted above, we next immunostained the tumor for additional markers that are variably present in MPNSTs (nestin, Sox10 and Sox2). A subset of tumor cells was immunoreactive for the intermediate filament nestin (30–70% of the cells in each field; Fig. 1d), much as was observed for the calcium binding protein S100β (30–70% positivity; Fig. 1e). In addition, a subset of tumor cells had nuclear immunoreactivity for the transcription factor Sox10 (30–50% positivity; Fig. 1g). Some cells in the tumor also demonstrated nuclear immunoreactivity for the immature/dedifferentiated Schwann cell marker Sox2 (20–40% positivity; Fig. 1h). No staining was observed in non-immune controls (Fig. 1f,i). These findings supported the diagnosis of an MPNST. However, careful examination of the patient showed no stigmata of NF1 such as dermal or plexiform neurofibromas, optic pathway gliomas, café-au-lait macules, axillary freckling, Lisch nodules, or bony dysplasia and there was no family history of NF1. There also was no history of radiotherapy prior to the development of the neoplasm. We therefore concluded that this neoplasm was a WHO grade IV sporadic MPNST.

**Figure 2:**
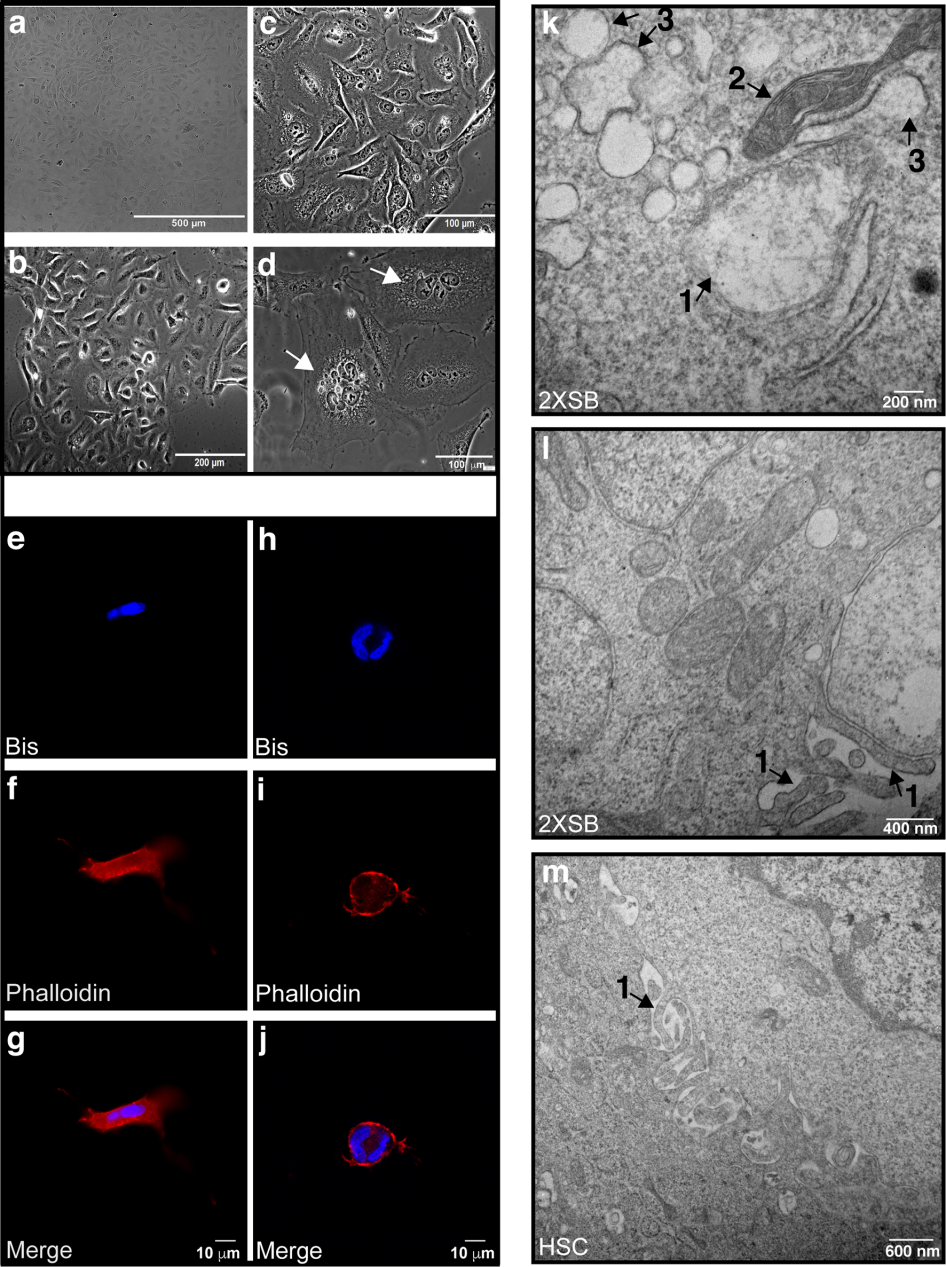
The morphology of the 2XSB sporadic MPNST cell line recapitulates the pattern seen in the parent tumor. (**a–d**) Representative brightfield (**a**) and phase contrast (**b**–**d**) images of logarithmic phase 2XSB cells. Both brightfield and phase contrast microscopy show that the tumor cells have a cobblestoned to spindled morphology. Note also that the ×60 phase contrast images in (**c**) show several cytoplasmic vacuoles in the tumor cells, while (**d**) illustrates two representatives of the subpopulation of large multinucleated cells present in this line (arrows). (**e–j**) Two representative 3D culture images (**g**,**j**) show the merged nuclei and cytoskeleton images acquired at ×63 with z-stack microscopy highlighting spindled (**e–g**) and polygonal (**h–j**) multinucleated cells. Panels (**e–i**) show the individual channels acquired for phallodin-Texas Red (cytoskeleton labeling, **f**,**i**) and DNA-bisbenzamide (nucleus labeling, **e**,**h**) prior to merging in panels (**g**) and (**j**). (**k–m**) Transmission electron microscopy (**k**) demonstrates that 2XSB cells contain swollen (1) and normal (2) mitochondria and swollen rough endoplasmic reticulum (3). Both 2XSB cells and non-neoplastic human Schwann cells also have numerous microvilli on their cellular surfaces (1 in **l**,**m**).

We established a cell line from this sporadic MPNST which we designated 2XSB. In conventional two-dimensional (2D) monolayer growth 2XSB cells had a spindled to polygonal morphology (Fig. 2a–d). High power examination showed that they often contained multiple cytoplasmic vacuoles (Fig. 2c). A subpopulation of cells that were larger and appeared to be multinucleated (Fig. 2d, arrows) were present in these cultures, as was seen in the parent tumor. To better visualize the multinucleated subpopulation, we fixed three-dimensional (3D) cultures of 2XSB cells grown on Matrigel 24-h after plating. We then stained these fixed cultures with the nuclear marker bisbenzimide and Texas red-conjugated phalloidin, a lectin that binds to actin. Confocal microscopy confirmed the presence of multinucleated cells with a spindled and polygonal morphologies (Fig. 2e–j, respectively). Thus, the 2XSB cell line contains heterogeneous subpopulations of mononuclear and multinucleated cells of varying sizes.

We next performed transmission electron microscopy (TEM) to examine the ultrastructural characteristics of 2XSB cells. We found that these cells contained both swollen (Fig. 2k, “1”) and normal mitochondria (Fig. 2k, “2”). In addition, swollen rough endoplasmic reticulum (RER) was readily identified (Fig. 2k, “3”), suggesting that the cells were experiencing ER stress. The cell membrane of 2XSB cells was focally studded with long microvilli (Fig. 2l, “1”). To determine whether microvilli were similarly present on the surface of non-neoplastic Schwann cells, we performed TEM on cultures of normal human Schwann cells. We found that non-neoplastic human Schwann cells similarly exhibited long microvilli on their cell surfaces (Fig. 2m, “1”). Microvilli are often associated with ion exchange functions and may be characteristic of cells derived from the Schwann cell lineage, as microvilli are present in normal Schwann cells in vivo adjacent to the node of Ranvier.

**Figure 3:**
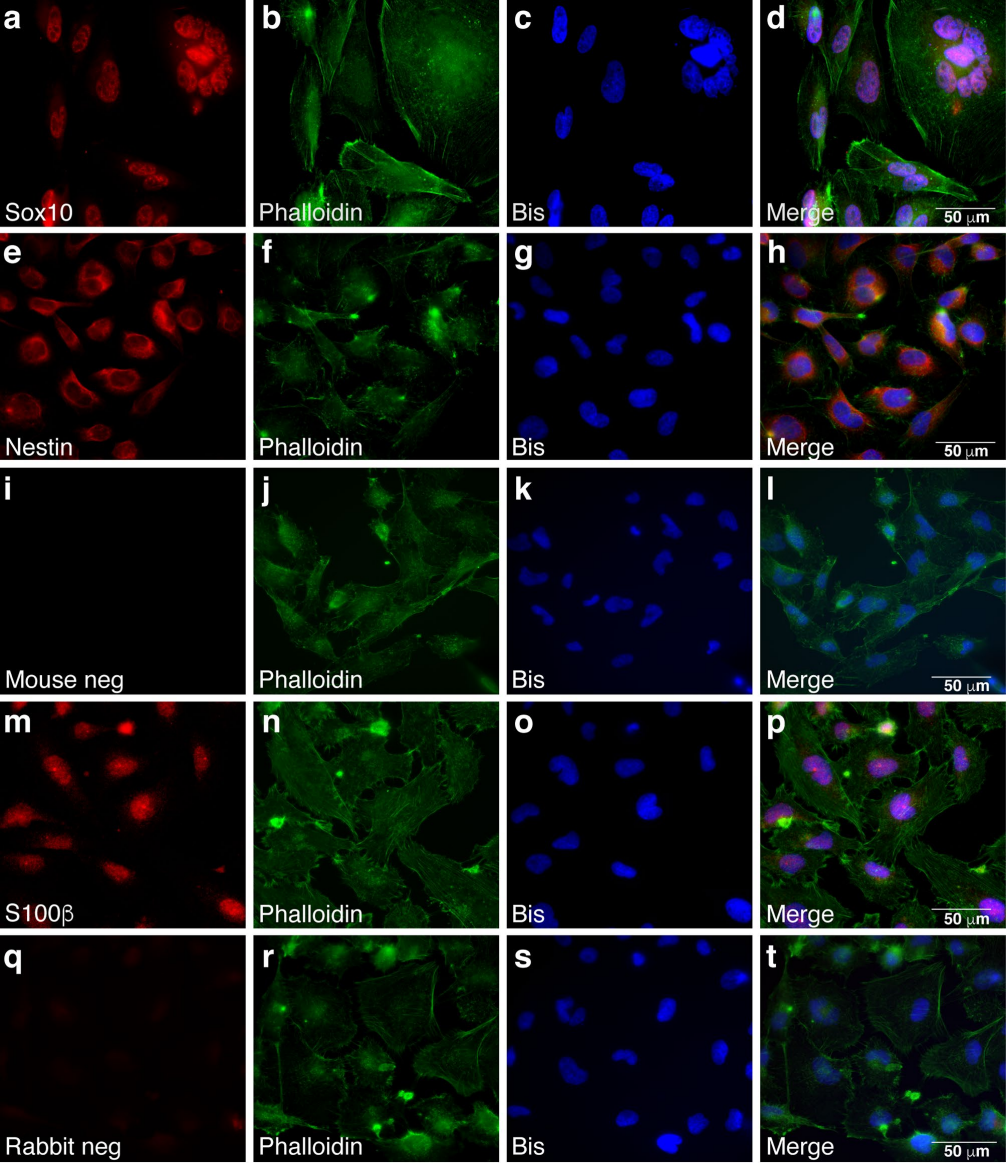
The immunoreactivity of the 2XSB sporadic MPNST cell line recapitulates the pattern seen in the parent tumor. (**a–h**,**m–p**) Representative immunofluorescence images showing Sox10 (a-d; red), nestin (**e**–**h**; red) and S100β (**m**–**p**; red) immunoreactivity in 2XSB cells. (**i**–**l**,**q**–**t)** Representative negative control images from preparations in which the primary antibody was replaced with non-immune mouse IgG or rabbit IgG are presented in (**i**–**l**) and (**q**–**t**), respectively. To highlight the morphology of these cells, preparations were simultaneously stained with the actin-binding lectin phalloidin (green channel). Bisbenzimide was used as a nuclear counterstain (blue channel).

To determine whether 2XSB cells maintained the immunophenotype of the parent tumor, we stained 2XSB cells for S100β, Sox10 and nestin. Like the parent tumor, 2XSB cells were immunoreactive for Sox10 (80–100% positivity; Fig. 3a–d), nestin (90–100% positivity; Fig. 3e–h) and S100β (80–100% positivity; Fig. 3m–p). Staining was abolished when the primary antibodies were replaced with non-immune mouse (Fig. 3i–l) or rabbit (Fig. 3q–t) IgG. We noted that the fraction of 2XSB cells labeling for these markers was higher than we observed in the parent tumor, suggesting either that our culture conditions selected for cellular subpopulations expressing these markers or that our culture conditions altered the expression of these markers. It has been previously reported that Sox2 expression is induced in mature non-neoplastic Schwann cells when they are cultured and that Sox2 expression is thus not necessarily indicative of immaturity in vitro^10^. Consequently, we did not examine Sox2 expression in 2XSB cells. We have carried 2XSB cells for > 80 passages without any change in the morphologic and immunohistochemical features described above.

We performed a short tandem repeat (STR) analysis to define a reference fingerprint for the 2XSB cell line. We then compared the STR profile of 2XSB cells to the STR profiles of the limited repertoire of sporadic MPNST lines (Hs-PSS^11^, Hs-Sch-2^11^ and STS-26T^12^ cells) that are currently available to demonstrate that the 2XSB STR profile was distinct from these other lines and to verify that it was not contaminated with cells from other lines held in our laboratory. The STR profile of 2XSB cells was distinct from that of the Hs-PSS, Hs-Sch-2 and STS-26 T sporadic MPNST cell lines (Table 1). A comparison of the STR profile of 2XSB cells to that of the 12 NF1-associated MPNST cell lines that are currently held in our laboratory also showed that the STR profile of 2XSB cells had no resemblance to the STR profiles of any of these NF1-associated MPNST cell lines.

**Table 1:** Short terminal repeat (STR) profile of the human 2XSB MPNST cell line compared to the STR profiles of the Hs-PSS, Hs-Sch-2 and STS-26T sporadic MPNST cell lines.

	2XSB	Hs-PSS	Hs-Sch-2	STS-26T
Amelogenin	X	X, Y	X	X
CSF1PO	15	10,12	11, 13	10, 13
D13S317	8	8, 10	11	9, 10
D16S539	11	10	11, 12	12, 13
D18S51	15	14, 15	13	17, 18
D19S433	14, 15.2	10.2, 12, 13	10.2, 14.2	14
D21S11	30, 30.2	28, 32	29, 30	31
D2S1338	17, 26	19	20	20
D3S1358	16	16	18	14, 20
D5S818	11	9, 11	13	11, 12
D7S820	8, 9	10, 12	11	8, 11
D8S1179	11, 13	14, 15, 19	14	13, 14
FGA	19, 23	22		22, 23
TH01	7, 9.3	6, 9	6, 7	6, 9.3
TPOX	11, 12	8	8	8
vWA	17, 18	14, 15	15, 16	17

**Figure 4:**
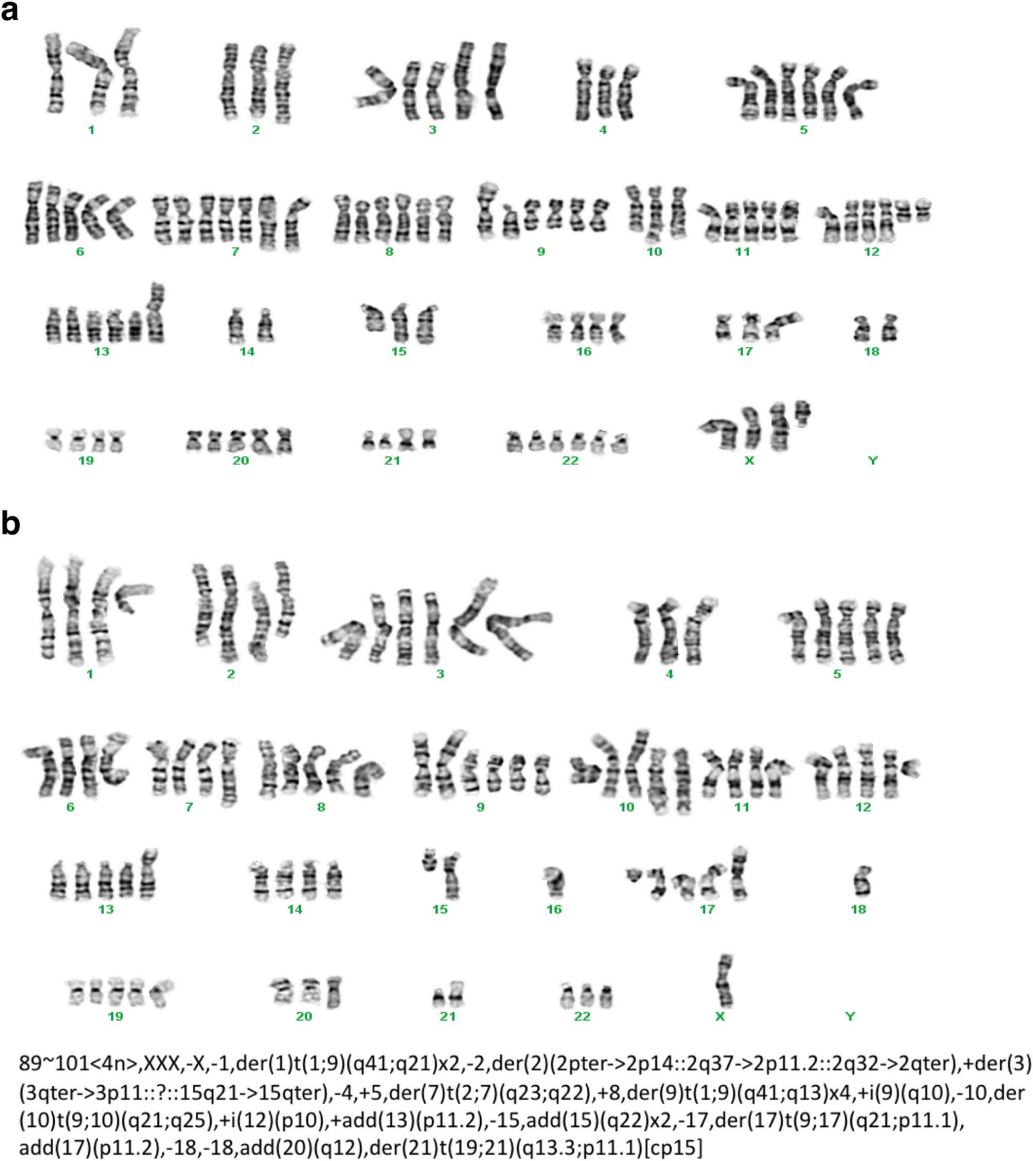
Karyotyping of the 2XSB sporadic MPNST cell line demonstrates the presence of two distinct populations with 89 (**a**) or 101 (**b**) chromosomes and numerous complex rearrangements of the tumor cell genome. Complex karyotyping can be characteristic of genetically unstable, highly aneuploid tumor types.

As an initial step towards characterizing the genomic abnormalities present in the 2XSB cell line, we examined the karyotype of these cells at passage 42. We found that there were two distinct karyotypes present in our 2XSB cultures. Neither of these karyotypes were diploid; one component had 89 chromosomes (Fig. 4a), while the other had 101 chromosomes (Fig. 4b). These karyotypes were highly complex, with numerous structural abnormalities evident in each subpopulation. A delineation of these structural variations indicated that the collective karyotype of our 2XSB cells was 89 ~ 101 < 4n > ,XXX,-X,-1,der(1)t(1;9)(q41;q21) × 2,-2,der(2)(2pter- > 2p14::2q37- > 2p11.2::2q32- > 2qter), + der(3)(3qter- > 3p11::?::15q21- > 15qter),-4, + 5,der(7)t(2;7)(q23;q22), + 8,der(9)t(1;9)(q41;q13) × 4, + i(9)(q10),-10,der(10)t(9;10)(q21;q25), + i(12)(p10), + add(13)(p11.2),-15,add(15)(q22) × 2,-17,der(17)t(9;17)(q21;p11.1),add(17)(p11.2),-18,-18,add(20)(q12),der(21) t(19;21)(q13.3;p11.1)[cp15]. These two karyotypes were present in approximately equivalent proportions in 2XSB cells and we found that the two karyotypes shared some similar chromosomal abnormalities (e.g., abnormal chromosomes 1, 9 and 15).

**Figure 5:**
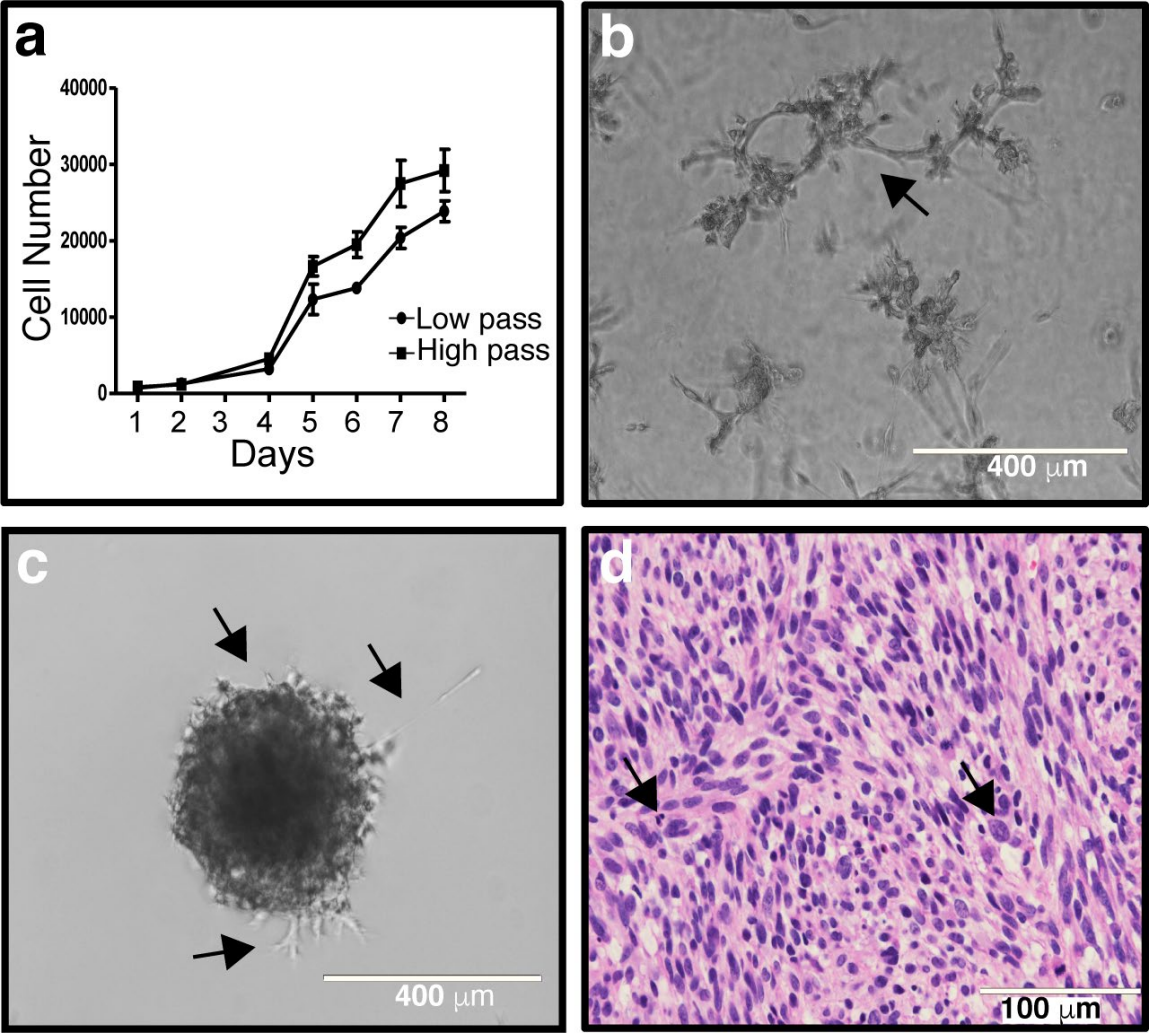
Growth characteristics of 2XSB MPNST cells in 2D cultures, 3D cultures, clonogenic assays and xenografts. (**a**) In vitro doubling time of both low (P6) and high passage (P54) cultures shows a consistent rate of proliferation in these passages. (**b**) Representative image of a 3D culture of 2XSB cells grown for 1-week post-plating on Matrigel demonstrating the high degree of invasive projections into Matrigel characteristic of this line (arrow). (**c**) Representative image of a clonogenic round bottom spheroid assay 1-week post-plating showing that 2XSB cells have the ability to survive and proliferate when embedded in Matrigel, resulting in the formation of colonies. These colonies also showed multiple regions of tumor cells invading the Matrigel (arrows). (**d**) A ×40 image of a hematoxylin and eosin stained section of a xenograft derived from 2XSB cells showing that the xenograft has histologic features resembling those of the parent tumor. Arrows indicate some of the numerous mitotic figures present in this xenograft.

To define the behavior of 2XSB cells in different in vitro and in vivo conditions, we assessed the doubling time of 2XSB cells in 2D monolayer cultures, their ability to maintain growth in 3D cultures, and their ability to establish xenografts in immunodeficient mice. We determined the doubling time of 2XSB cells both immediately after the withdrawal of NRG1β and forskolin (passage 6) and at a much later passage (passage 54). We found that the doubling times of 2XSB cells were similar at both time points; the doubling time at passage 6 was 28.0 ± 3.09 h, while the doubling time at passage 54 was 27.3 ± 4.06 h (Fig. 5a). We also observed their cell division dynamics using live cell microscopy. Consistent with our immunocytochemistry observations, we observed cells of varying size with both single and multiple nuclei. These cells were highly mobile and demonstrated both successful and abnormal cytokinesis. We captured the division of one of the mononuclear cells into two cells as well as the division of a small, single-nucleated cell into three cells, suggestive of abnormal cytokinesis (see 00:35–42 and 00:58–01:25 in Supplementary Video 1, respectively). We also observed delayed cell division kinetics with the large multinucleated cells (Supplementary Video 2) and, on occasion, observed the division of a large multinucleated cell into multiple cells (cell in lower right corner of Supplementary Video 3).

We established 3D spheriod cultures to determine whether 2XSB cells could attach to and invade Matrigel, which is composed largely of the basement membrane proteins collagen type IV and laminin. We found that 2XSB cells attached to a Matrigel surface and that, by 1-week post-plating (Fig. 5b), > 90% of the 2XSB colonies had aggressively invaded the Matrigel. In 3D clonogenic embedded culture assays, 2XSB cells also readily formed colonies in Matrigel, indicating that they were capable of clonogenic survival and proliferation (Fig. 5c); we observed an invasive growth phenotype from these colonies as well (Fig. 5c, arrows). Next, we assessed the 3D invasive potential of giant cells isolated from the parental line. We found that the giant cells had an invasive phenotype similar to that of the parent culture (Fig. 5b) when plated in 3D cultures (Supplementary Fig. S2a–c). Finally, we subcutaneously injected NOD.*Cg-Prkdc*^*scid*^*Il2rg*^*tm1Wjl*^*/Szj* (NOD-Scidγ) mice with 3 × 10^6^ 2XSB cells in 50% Matrigel. Although these xenografts grew slowly in vivo (11 weeks to reach 1 cm in diameter, Supplementary Fig. S2d), they did form tumors in NOD-Scidγ mice. Microscopic examination showed that xenografts established from 2XSB cells were composed of cells with a morphology similar to that of the parent tumor (Fig. 5d).

### Genomic analyses indicate that 2XSB cells largely recapitulate the abnormalities seen in the parent tumor

It is widely appreciated that cell lines established in vitro can be derived solely from tumor subpopulations that adapt well to culture conditions and thus may not represent the full spectrum of genomic abnormalities present in the parent tumor. To determine whether this was the case with 2XSB cells, we compared the genomic abnormalities in 2XSB cells to those in its parent tumor using high density single nucleotide polymorphism (SNP) arrays. High-density SNP array analysis of the parent tumor showed copy number gains spanning most of chromosomes 2, 5, 6, 7, 8, 11, 17 and 19, with large regions of gain also present that involved distal 1q, 3q, 4q, 9q, 10q, 12p, 13q, 15q, 16q, proximal 20p, 20q, 21q, 22q and the proximal part of Xp and Xq (Supplementary Figs. S3a and S4a). Focal gains were present in 1p, the centromeric region of chromosome 3, 4q, 11p, two different sites in 11q, proximal 17q, 18p, 18q, proximal 18q and 19p; some of these focal gains were superimposed on more extensive regions of gain. Large regions of chromosomal loss were less common, affecting chromosomes 10p, 18p, distal 18q and Xq, with focal losses evident in chromosomes 5p, 8p, 9p, and Xq. A comparison of the parent tumor SNP array profile to that of 2XSB cells showed that these two profiles were nearly identical (compare Supplementary Figs. S3a,b, S4a,b). Nonetheless, some minor differences were evident. 2XSB cells lacked the focal 1q, 4q, 11p, 16p, 16q, 17q and 19p gains and the 5p loss, one of the 8p focal losses, the 10p focal loss, the Xp arm loss, and the focal Xq loss that were evident in the parent tumor. In addition, the region of gain on chromosome 9 was more extensive in the 2XSB cell line than in the parent tumor. The only other genomic change detected solely in the 2XSB cell line was a focal copy number gain in proximal 18q, just distal to the shared region of 18q gain observed in both the parent tumor and 2XSB cells.

### High density SNP array of 2XSB cells shows alterations in some genes classically associated with MPNST pathogenesis

**Figure 6:**
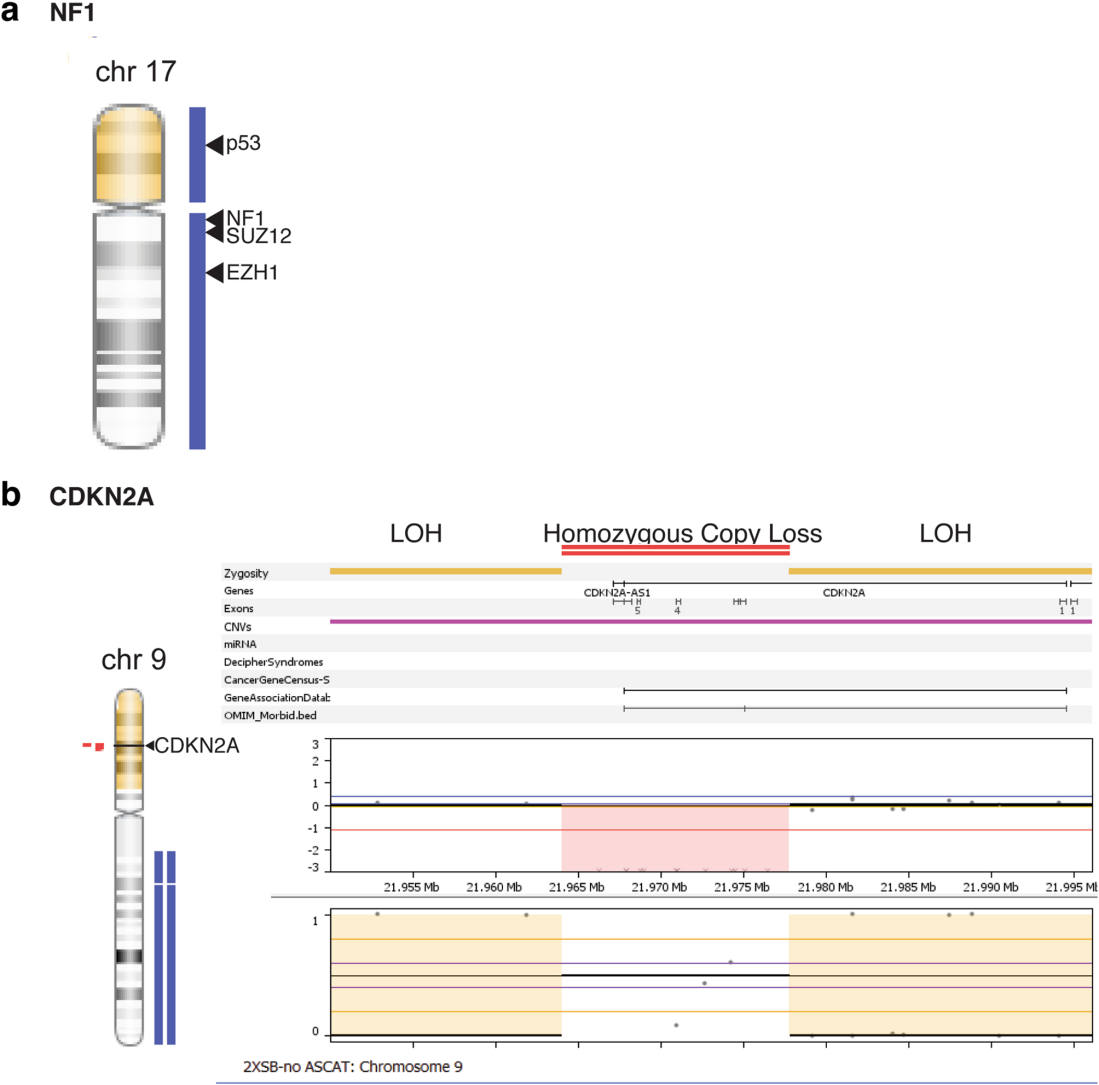
SNP array copy number analysis of the *NF1* (**a**) and *CDKN2A* (**b**) genes in 2XSB cells. Although no losses are evident in *NF1*, homozygous deletions are present in *CDKN2A*.

We examined the high-density SNP array data to determine whether 2XSB cells had deletions in tumor suppressor genes commonly mutated in NF1-associated MPNSTs. The tumor suppressors *TP53* and *NF1* are located at p13.1 and q11.2, respectively, of chromosome 17. 2XSB cells showed no evidence of genetic deletions on chromosome 17 but did have two large regions of copy number gain (Fig. 6a). The first of these gains (0–22,242,355) with loss of heterozygosity (LOH) included the entire *TP53* gene (7,565,097–7,590,856), while the second region of gain (25,295,032–30,784,881) included the entire *NF1* (25,295,032–81,195,210), *EZH1* (40,852,293–40,897,071) and *SUZ12* (25,295,032–81,195,210) genes (Supplementary Table 1). Similar copy number alterations were also observed in the parent tumor (Supplementary Fig. S5a, Supplementary Table 1), although the observed copy number gain did not encompass the entire *NF1* gene. We also observed copy number gains in *EZH2* (chr7:148,504,464–148,581,441), *PTEN* (with LOH, chr10:89,622,870–89,731,687), and *EED* (chr11:85,955,806–85,989,785) in both the cell line and tumor (Supplementary Table 1) Like *NF1*, the tumor suppressor *CDKN2A* is frequently mutated in NF1-associated MPNSTs. Homozygous deletions of *CDKN2A* (9p21.3) were present in 2XSB cells (Fig. 6b, Supplementary Table 1). The *CDKN2A* deletions detected in 2XSB cells was also present in the parent tumor (Supplementary Fig. S5b, Supplementary Table 1). No additional deletions were detected in genes encoding other cell cycle regulatory proteins [CDKN2B (p15), CDKN2C (p18), CDKN2D (p19), CDKN1A (p21), CDKN1B (p27), CDKN1C (p57), CDK2 (CDKN2, p33)]; indeed, several of these genes had copy number gains or loss of heterozygosity (LOH).

### Whole exome and targeted sequencing of 2XSB cells identifies candidate pathogenic mutations

In addition to *NF1*, *TP53*, *CDKN2A*, *PTEN,* and genes encoding components of PRC2, a number of other genes have also been suggested to contribute to the pathogenesis of MPNSTs. These genes include *BRD4*^23^, *BIRC5*/survivin^24^, *COX2*^13^, *EGFR*^14^, *EFNA3*^15^, *ERBB2*^16^, *EYA4A*^17^, *HIF1A*^18^, *MAF*^19^, LATS1/2^20^, *MEIS1*^21^, *RASSF1A*^25^, *SOX9*^26^, *STAT3*^18^, *STAT5*^23^, *TAGLN*^24^, *TCTP*^27^, *TOP2A*^28^, *TYK2*^29^, *WNT*^30^, and *WT1*^31^. In addition to these genes, a much larger cohort of genes has been linked to the pathogenesis of other human cancer types. At the time of this study, the Bushman Laboratory Cancer Gene List (http://www.bushmanlab.org/links/genelists) included 2027 genes that play a role in the development of human cancers; this list was compiled from multiple sources including the Atlas of Genetics and Cytogenetics in Oncology and Hematology^32^, CANgenes^33^, CIS^34^ and COSMIC^35^. Consequently, we performed whole exome sequencing (WES) of 2XSB cells and their parent tumor and asked whether they contained pathogenic mutations in any of the genes previously implicated in the pathogenesis of NF1-associated MPNSTs or included in the Bushman Laboratory Cancer Gene List.

Our WES workflow is outlined and summarized in Supplementary Fig. S6. We processed our FASTQ files through two separate alignment and variant callers (DNAStar and Varsome). In 2XSB cells, 2989 non-synonymous variants representing 2058 genes were present. We then focused on variants affecting genes in the Bushman Laboratory Cancer Gene List and genes previously implicated in MPNST pathogenesis (see Supplementary Table 2 for a complete listing of the genes we examined). This yielded 326 variants in 196 genes (Supplementary Fig. S6).

**Figure 7:**
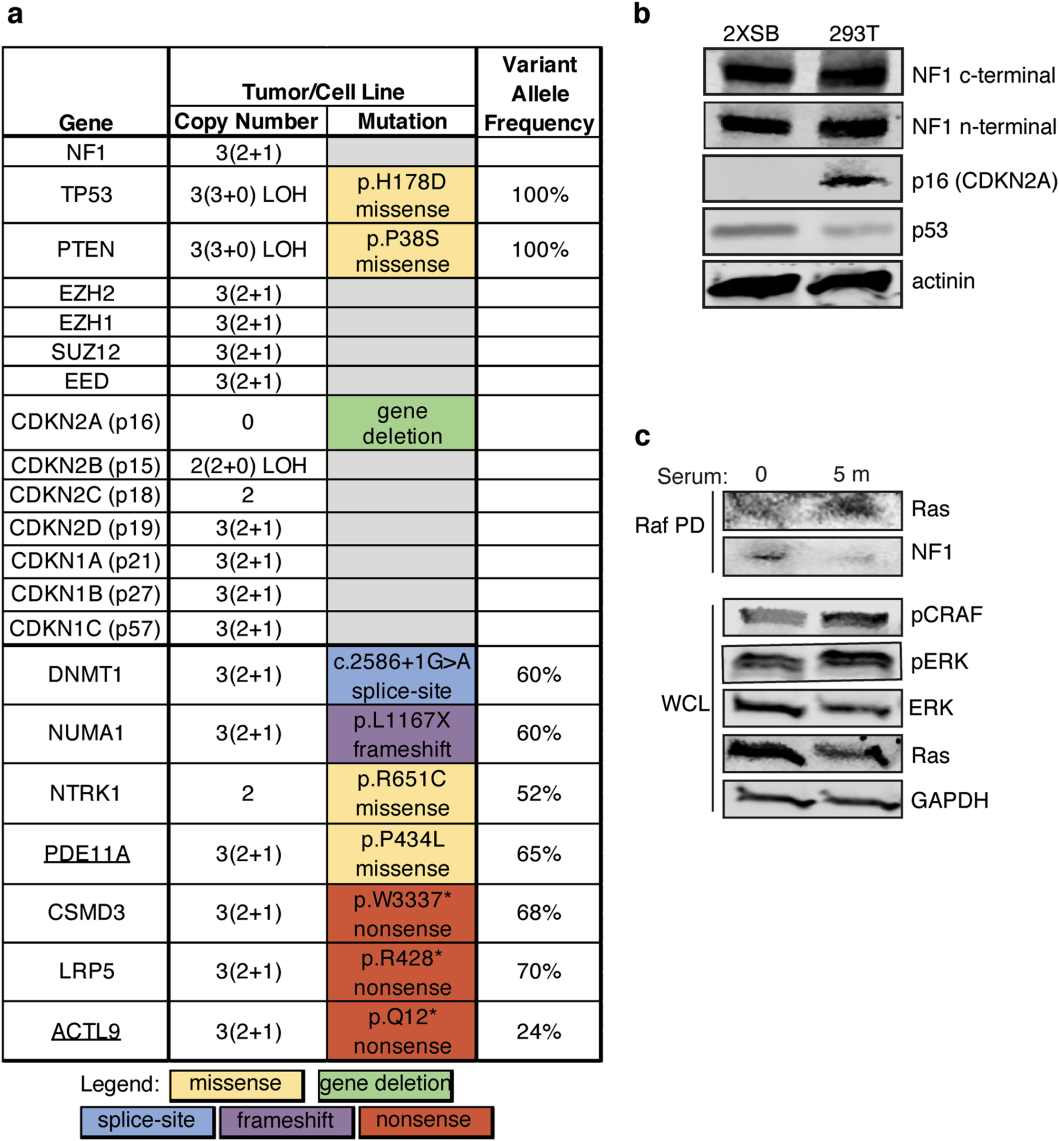
Mutational and functional status of key genes in 2XSB cells. (**a**) Summary of mutations identified by SNP array and/or WES sequencing analyses in genes previously implicated in MPNSTs and/or included in the Bushman Laboratory Cancer Gene List. Gene names that are underlined represent likely pathogenic calls and all others are pathogenic mutations. For copy number changes, the numbers in parentheses indicate the copy number of each haplotype, while the numbers outside the parentheses indicate the total copy number. If a mutation was not identified in a gene, the box in the mutation column is gray. (**b**) Immunoblot blot analysis of cell line-derived whole cell lysates from 2XSB cells and a control cell line (293 T). Lysates were prepared from 2D cultures of log phase cells and were probed with anti-C-terminal NF1, anti-N-terminal NF1, anti-CDKN2A (p16), and anti-p53 antibodies. Actinin was used as a loading control. (**c**) Ras activation assays were performed to determine the functional status of NF1 in serum deprived 2XSB cells stimulated with 10% serum for 5 min. Activated Ras was identified by Raf pull-down (Raf PD) with the Raf-Ras binding domain beads followed by immunoblotting with pan-Ras and NF1 antibodies. Activation of downstream effectors of Ras was determined in whole cell lysates by immunoblot analyses with anti-pCRAF and -pERK antibodies. Lysates were also probed with anti-total ERK and pan-Ras antibodies. GAPDH was used as a loading control.

268 of the 326 variants were classified as benign, 50 as being of uncertain significance and 10 were pathogenic (8) or likely pathogenic (2). The 10 pathogenic/likely pathogenic variants included 1 splice-site mutation, 1 frameshift mutation, 3 nonsense mutations and 5 missense mutations (Supplementary Fig. S6); these variant calls were manually evaluated for quality control after processing through the two independent sequence read aligners and cancer variant callers (Supplementary Fig. S7, Supplementary Table 4). We found no evidence of point mutations in *NF1, SUZ12* or in any of the genes encoding other PRC2 (*EED*, *EZH1* and *EZH2*) components in either the 2XSB cell line or the parent tumor (Fig. 7a). Other than the *CDKN2A* homozygous gene deletion, we did not detect mutations in any of the other CDKN1 or CDKN2 (*CDKN1A/B/C*, *CDKN2B/C/D*) genes (Fig. 7a). However, we did identify a pathogenic *TP53* mutation, p.H178D (Fig. 7a), in both the cell line and parent tumor.

Of the remaining 9 pathogenic/likely pathogenic mutations, one was in *PTEN,* a gene that is frequently mutated in NF1-associated MPNSTs. As noted above, we observed an unbalanced copy number gain affecting the region of chromosome 10 (nucleotides 42,356,527–135,534,747) that includes *PTEN* in both 2XSB cells and the parent tumor (Supplementary Table 1); this LOH was accompanied by the presence of a pathogenic missense mutation in *PTEN*, p.P38S (Fig. 7a). The remaining pathogenic/likely pathogenic mutations detected included three nonsense (stop-gained) variants in *CSMD3* (p.W3337*), *LRP5* (p.R428*), and *ACTL9* (p.Q12*)*;* one frameshift variant in *NUMA1* (p.L1167X)*;* three missense variants affecting *NTRK1* (p.R651C), *PDE11A* (p.P434L), and *BRAF* (p.V600E)*;* and one splice-site mutation in *DNMT1* (c.2586 + 1 G > A). Aside from *BRAF*^36^, none of these genes have been previously implicated in MPNST pathogenesis. All of these variant calls were congruent between the two callers.

*TERT* promoter mutations have been previously reported in a small subset of MPNSTs^37–39^, with some investigators suggesting that *TERT* promoter mutations are more common in sporadic MPNSTs than NF1-associated MPNSTs^38^. In addition, previous studies have indicated that *TERT* is highly expressed in many high grade and some low grade MPNSTs^40,41^. Since the *TERT* promoter is not routinely captured in WES, we used PCR to amplify a 306 bp region of the *TERT* promoter^42^ from 2XSB cells, their parent tumor, wild-type Schwann cells and 10 additional MPNST cell lines. These PCR products underwent Sanger sequencing to identify any known cancer-associated mutations in the *TERT* promoter. Both the parent tumor and 2XSB cells had a common mutation in the core *TERT* promoter, C250T (− 146 bp; Supplementary Fig. S8a,b). Four of the other ten MPNST lines (ST88-14, Hs-PSS, T265-2c and NSF1) had the most common C228T (− 124 bp) TERT promoter mutation, while four others (MPNST2, NMS2-PC, NMS2, Hs-PSS) had the T349G (− 245 bp) mutation (Supplementary Fig. S8c,d). The Hs-PSS cell line, another sporadic MPNST line, was the only one to have both the C228T and the T349G mutations. These mutations were all absent in the wild-type human Schwann cells. The presence of the C250T mutation in 2XSB cells led us to ask whether *TERT* mRNA was highly expressed in 2XSB cells and the parent tumor compared to wild-type Schwann cells. Despite the presence of the *TERT* promoter mutation, *TERT* transcripts were difficult to detect in 2XSB cells and the parent tumor (Supplementary Fig. S1).

Our genomic analyses indicated that the *NF1* gene was intact in 2XSB cells. To confirm that this line expresses full length NF1 protein (neurofibromin), we performed immunoblot analyses with antibodies recognizing either the N-terminus or the C-terminus of this protein. Both antibodies detected full-length neurofibromin protein in 2XSB cells (Fig. 7b), indicating that 2XSB cells are not *NF1*-null. In keeping with our finding of homozygous *CDKN2A* gene deletion, p16 protein was undetectable by immunoblot analysis (Fig. 7b). Immunoblot analyses did show p53 expression in 2XSB cells (Fig. 7b). We conclude that *TP53* is mutated and expressed in 2XSB cells, as is commonly seen in NF1-associated MPNSTs.

To confirm that the neurofibromin expressed by 2XSB cells is functional, we performed Ras activation assays. In these assays, we stimulated serum-deprived 2XSB cells with 10% serum for 5 min, as these conditions induce peak neurofibromin degradation and Ras-GTP activation^43^. We found that serum stimulation resulted in an increase in Ras activation (Fig. 7c) compared to unstimulated cells. We also observed increased phosphorylation of the downstream Ras effector molecules CRAF and ERK in serum-stimulated cells compared to untreated 2XSB cells (Fig. 7c, Supplementary Fig. S9). These data indicate that neurofibromin is functional in 2XSB cell lines and that it represses Ras activation and Ras-mediated signaling events in a context-dependent manner.

## Discussion

The nerve-associated tumor from which we derived 2XSB cells was diagnosed as an MPNST after eliminating other diagnostic possibilities and its identification as an MPNST was supported by histologic, immunohistochemical and genomic analyses. The patient from which the tumor was resected had no previous diagnosis of any malignancy, lacked the clinical features characteristic of NF1 and had no previous history of radiotherapy, leading us to conclude that this tumor was a sporadic MPNST. High-density SNP array and whole exome sequencing analyses showed no evidence of inactivating *NF1* mutations in either 2XSB cells or their parent tumor and we demonstrated that 2XSB cells expressed functional full-length neurofibromin protein, indicating that 2XSB cells are derived from the subset of sporadic MPNSTs that have intact *NF1*. As the pathogenesis of *NF1*-intact sporadic MPNSTs is poorly understood, the 2XSB cell line represents a valuable tool for investigating the mechanisms underlying the pathogenesis of neurofibromin-expressing sporadic MPNSTs. However, the distinct subpopulations of Sox2-positive cells and polyploid cells that we identified raise important questions about the biology of sporadic MPNSTs and their response to therapeutic agents. Although we recognize the risks of generalizing findings from one cell line, the genomic abnormalities that we identified in 2XSB cells may also point to some of the signaling pathways that will be important therapeutic targets in neurofibromin-positive sporadic MPNSTs.

We identified two cellular subpopulations in the 2XSB parent tumor that potentially play an important role in the growth of sporadic MPNSTs and resistance to chemotherapy. The first of these subpopulations is the cells that are immunoreactive for Sox2, a transcription factor that is expressed in immature and dedifferentiated Schwann cells^44^ and self-renewing progenitor cells in the central nervous system^45^. Sox2, when transfected together with vectors expressing Oct4, c-Myc and Klf4, can reprogram mature cells into an embryonic pluripotent state^33^; in many cancer types, Sox2 also plays an important role in maintaining cancer stem-like cells which promote clonogenicity, cancer cell mobility, metastasis, relapse and resistance to chemotherapy^46^. This raises the question of whether the subset of Sox2-positive cells in the 2XSB tumor are cancer stem-like cells or simply a subpopulation with features similar to immature or dedifferentiated Schwann cells. In future studies, it will be important to determine whether the Sox2-positive subset of 2XSB cells are capable of continuous self-renewal, demonstrate the ability to differentiate along multiple lineages and establish grafts with greater efficiency than Sox2-negative 2XSB cells.

The second subpopulation of special interest in the 2XSB cell line and its parent tumor is the multinucleated cells. The presence of giant polyploid cells has been previously reported in MPNSTs (e.g. Ref.^47^). Given the extensive genomic abnormalities we identified in 2XSB cells, we considered the possibility that these multinucleated cells were a residual subpopulation that had accumulated so much genetic damage that they were no longer capable of cellular division. However, the fact that these polyploid cells persisted through more than 80 passages indicated that they are capable of replication. Further, our live cell imaging and 3D culture analyses indicated that these multinucleate cells continue to divide and are invasive. This led us to consider the possibility that the multinucleated cells form as a result of endoreplication and failed cytokinesis. In other cancer types, polyploid giant cancer cells (PGCC) can form by endomitosis (mitosis followed by failed cytokinesis). Endocycling (endoreplication of the genome without entering mitosis) can also result in the production of polyploid cells. Both of these phenomena can co-exist in cancer cells^48^. A third possibility is that the multinucleated cells form by fusion of uninuclear cells. At present, we do not know which of these mechanisms drives the formation of multinucleated 2XSB cells. However, the fact that 2XSB cells have pathogenic *TP53* and *CDKN2A* mutations makes us suspect that endocycling is responsible for the formation of this 2XSB subpopulation. The presence of these cells may also have clinical relevance since PGCCs in other cancers promote drug resistance under conditions of physiological or pathological stress (e.g. in cancers receiving radiotherapy)^49–51^. In future studies, it will be interesting to determine what mechanisms promote the production of polyploid 2XSB cells and whether this subpopulation is particularly resistant to therapeutic agents.

The genomes of 2XSB cells and their parent tumor are aneuploid with extensive chromosomal breaks and rearrangements. In both 2XSB cells and the parent tumor, copy number gains predominated, with losses being less common. Previous studies have similarly indicated that highly variable chromosomal gains, losses and rearrangements are common in NF1-associated MPNSTs^52^. Despite this variability, some common patterns have been identified in the genomes of MPNSTs. MPNSTs often are hypodiploid or near triploid, with gains most often occurring in chromosome 7, 8q and 15q and losses evident in chromosomes 1p, 9p, 11, 12p, 14q, 17q, 18, 22q, X and Y^53–61^. In 2XSB cells and their parent tumor, we identified gains of chromosomes 7, 8 and 15q, which is consistent with these earlier studies. In keeping with previous cytogenetic analyses of MPNSTs, we also identified losses of 18p, distal 18q and Xq. We did not find losses in 2XSB cells of the other chromosomal regions previously reported to be commonly lost in MPNSTs. However, given the variability previously observed in the genotypes of other MPNSTs, this is not surprising.

Previous studies have indicated that mutations affecting *NF1*, *TP53*, *CDKN2A*, *PTEN* and genes encoding components of PRC2 are the most common mutations that occur in MPNSTs regardless of whether they are NF1-associated, radiation-induced or sporadic^62–66^. Although *NF1* and genes encoding PRC2 components are intact in 2XSB cells, these cells did have mutations in *TP53*, *CDKN2A* and *PTEN*, three genes which have been previously found to be mutated in both NF1-associated and sporadic MPNSTs^1,67–70^. The *TP53* (c.532C > G, exon 5, H178D; LOH) mutation [previously found in breast, genital tract, ovary, endometrial and lung cancers (COSMIC)] and homozygous *CDKN2A* loss that we identified in 2XSB cells promote genomic instability and loss of cell cycle checkpoints in other cancer types. Similarly, the *PTEN* mutation (c.112C > T in exon 2, p.P38S; LOH) that we found in 2XSB cells is a known pathogenic mutation previously identified in melanomas and endometrial carcinomas (COSMIC). It is thus highly likely that *TP53*, *CDKN2A* and *PTEN* are driver genes in 2XSB cells and that dysregulation of the cell cycle and phosphatidylinositol 3-kinase (PI3K) signaling played an important role in the development of this sporadic MPNST, much as it does in NF1-associated MPNSTs. A pathogenic *BRAF* mutation (c.1799T > A, p.V600E) and copy number gain (2 + 1) was also detected in the tumor and derived cell line. Although its function in this setting has not been explored, this *BRAF* mutation has been previously reported in NF1-associated and sporadic MPNSTs^71^. The allelic balance of this mutation in our WES data suggests that 2XSB cells carry one copy of the mutant *BRAF* allele.

We also identified pathogenic or likely pathogenic mutations in seven genes that have not been previously implicated in MPNST pathogenesis. Intriguingly, these genes have diverse functions. *DNMT1* (DNA methyltransferase 1) is an epigenetic modifier that plays a key role in maintaining methylation patterns after DNA replication, which results in gene silencing in undifferentiated embryonic stem cells. *DNMT1* is also mutated in many cancer types. Given the importance of PRC2 component mutations in other MPNSTs, it is tempting to speculate that mutations in the *DNMT1* epigenetic modifier may have effects in 2XSB cells analogous to those produced by PRC2 mutations in other MPNSTs. *NUMA1* (nuclear mitotic apparatus protein 1; 3499–3500T < *, p.1167X) is a nuclear matrix protein that interacts with microtubules and plays a role in mitotic spindle formation^72^ that is required for cells to complete mitosis. *PDE11A* (phosphodiesterase 11A; c.1301C > T, p.P434L) is a second messenger that downregulates cAMP and cGMP signaling, two key signaling mediators frequently associated with calcium mediated signaling. Mutations in this gene occur in Cushing’s disease and testicular, adrenocortical, germ cell, prostate and pituitary tumors (cancerindex.org). *CSMD3* (CUB and Sushi multiple domains 3; 10010G < A p.N2621H) is normally highly expressed in the brain. *CSMD3* has been proposed as a candidate gene for autism^73^ and familial myoclonic epilepsy^74^ and may play a role in cell–cell adhesion. Nonsynonymous mutations in *CSMD3* are linked to familial colorectal cancer^75^ and basal cell carcinoma (COSMIC). *LRP5* (LDL receptor related protein 5; 1282C < T, p.R428*) is a transmembrane low-density lipoprotein receptor; it also interacts with Frizzled proteins to form a co-receptor that transduces signals by Wnt proteins^76^. The mutation that we identified in 2XSB cells is quite rare and has been implicated in the pathogenesis of osteoporosis-pseudoglioma syndrome (OPPG)^76,77^. *NTRK1* (neurotrophic receptor tyrosine kinase 1, also known as trkA; 1951C < T, p.R651C) is a receptor tyrosine kinase that, when activated by its ligand nerve growth factor, activates Ras, PI3K and other cytoplasmic signaling pathways. Although point mutations in *NTRK1* have not been previously reported in MPNSTs, *NTRK1* translocations have been seen in MPNSTs^78,79^. Finally, we identified a nonsense mutation in *ACTL9* (Actin Like 9; c.34C > T, p.Q12*) in 2XSB cells. Very little is known about this gene and it has weak associations with different cancer types. Although these findings are intriguing, we would caution that validation of these genes as driver genes in 2XSB cells will require additional functional studies. Given the heterogeneity of MPNSTs, we would also note that it is premature to conclude that these seven genes will prove to be driver genes in all neurofibromin-positive sporadic MPNSTs. In future studies, it will be necessary to define the functional role(s) of these genes by manipulating their expression in 2XSB cells and other MPNST cell lines. It will also be important to determine how frequently they are mutated in NF1-associated, radiation-induced and sporadic MPNSTs.

On another cautionary note, we would point out that some of the potentially pathogenic mutations we identified did not appear to have a functional impact. As an example of this, despite the fact that 2XSB cells and their parent tumor had a C250T (− 146 bp) mutation in the core promoter of the *TERT* gene, our RNA-Seq dataset showed very low *TERT* mRNA expression. This argues that this mutation has very little impact on *TERT* expression in 2XSB cells, despite the fact that this mutation alters *TERT* expression in other cancer types. At the same time, we are not trying to generalize the observations we made in 2XSB cells to other MPNSTs. We did identify *TERT* promoter mutations in several other MPNST cell lines and previous studies have indicated that *TERT* is highly expressed in many MPNSTs^40,41^. We would simply caution that *TERT* promoter mutations, even known pathogenic ones such as we identified in 2XB cells, are not necessarily predictive of increased *TERT* expression and telomerase activity. It is also not clear yet that high levels of *TERT* expression are invariably associated with *TERT* promoter mutations in MPNSTs. In future studies, the relationship between promoter mutations, increased *TERT* expression and telomerase activity in MPNSTs should be examined to better predict which tumors are most likely to have promoter mutations that result in increased telomerase activity.

It was also striking that we found gains rather than losses of the *NF1* gene in 2XSB cells. Although we initially suspected that these gains might subtly impact the structure of the NF1 locus, our immunoblots showed no evidence of atypically sized neurofibromin protein and the neurofibromin expressed in 2XSB cells was functional. This led us to consider instead whether these gains might result in overexpression of neurofibromin that chronically suppressed Ras activation (an action potentially partially offset by the presence of the *BRAF* V600E mutation). Again, though, we found no evidence that *NF1* was overexpressed in 2XSB cells and Ras was readily activated in response to serum stimulation. Consequently, our evidence thus far indicates that *NF1* gains in 2XSB cells have little functional impact.

In summary, we have derived a sporadic MPNST cell line that recapitulates the morphologic, immunohistochemical, and genomic abnormalities of the tumor from which it was derived. We have shown that this line grows effectively in a variety of culture conditions and as xenografts in immunodeficient mice. Intriguingly, 2XSB cells have intact *NF1* genes and thus are representative of the subclass of sporadic MPNSTs that differ in this regard from NF1-associated MPNSTs. Considered together, these features indicate that 2XSB cells will be a highly useful tool for deciphering the biology of sporadic MPNSTs and developing effective new treatments for these aggressive neoplasms.

## Methods

### Human studies

These studies were reviewed and approved by the Institutional Review Boards for Human Use of the University of Alabama at Birmingham and the Medical University of South Carolina. Human tissues used were excess material removed during standard therapy and were de-identified; consequently, neither IRB required informed consent. None of the specimens were from subjects under 18 years of age. All methods using human tissues were carried out in accordance with relevant guidelines and regulations.

### Antibodies and other reagents

The conditions utilized and the source of the primary antibodies used in this study are detailed in Supplementary Table 3. Secondary antibodies were purchased from ThermoFisher (Alexa Fluor Dyes) and Licor (IRDye8000CW and IRDye680RD). Non-immune primary ChromPure IgG antibodies were from Jackson ImmunoResearch (West Grove, PA). DAB kits were purchased from Vector Labs (#SK-4100).

### Establishment of the 2XSB sporadic MPNST cell line

Fresh tumor tissue was collected from the operating room in ice-cold phosphate buffered saline (PBS). After mechanical and enzymatic dissociation, the tissue was placed in poly-l-lysine/laminin coated tissue culture plates containing DMEM supplemented with 10% fetal calf serum, 10 nM NRG1β (a Schwann cell mitogen), 2 μM forskolin, 1% glutamine, 10 μg/mL streptomycin and 10 IU/mL penicillin. The tumor tissue was minced into 2–4 mm cubes, triturated and maintained for 48–72 h at 37 °C in 5% CO_2_ to allow cells to migrate out of the tissue. After 72 h, the remaining non-dispersed tissue was rinsed with PBS and then trypsinized for 15 min at 37 °C. An equal volume of DMEM with 10% fetal calf serum was added and dissociated cells were pelleted by centrifugation for 5 min at 5000 RPM. These cells were then resuspended and added to the cells that had previously migrated out of the tissue cubes. We allowed this culture to proliferate until confluent, with fresh media being added every 3–4 days. Confluent plates were trypsinized and split 1:2. After 5 passages, neuregulin-1β and forskolin were removed from the media and cells were maintained in DMEM supplemented with 10% fetal calf serum, 1% glutamine, 10 μg/mL streptomycin and 10 IU/mL penicillin.

### 3D cell culture

Cells were maintained at subconfluent log phase state in full growth media. Thawed Matrigel was added to each well (35 μL) of an 8-well chamber slide to form a bottom layer. After plating 2,500 2XSB cells in 400 μL of 2% Matrigel-culture media on top of the bottom layer, chamber slides were incubated at 37° in 5% CO_2_. Cells were fed every 2 days with 2% Matrigel growth media. They were imaged every 2 days, with the final images collected 10 days post-plating. For multinucleated visualization, 12,000 cells were plated in a 4-well chamber slide on top of 90 μL of 100% Matrigel and fixed the next day. 3D cultures were fixed with 4% paraformaldehyde for 20 min, washed 3× with PBS, permeabilized for 30 min, washed, stained with 400 μL of 5 μg/mL Hoechst 33342 (bisbenzimide, Thermo 62249) and 0.125 mg/mL Phalloidin (Invitrogen Texas Red-X #T7471) for 1 h washed and then mounted with Prolong Glass (Invitrogen; P36980).

### Karyotyping

G-banded chromosome analyses were performed on metaphase preparations of 2XSB cells using standard techniques. The G-banded karyotypes were interpreted according to the International System for Human Cytogenetic Nomenclature ISCN 2009^47^.

### Short tandem repeat profiling of sporadic MPNST cell lines

Short tandem repeat profiles were determined by examining 15 markers standardly used by the American Type Culture Collection and the amelogenin locus (included to verify the sex of the patient from which the tumor was derived) with an AmpFISTR system (Applied Biosystems; Foster City, CA).

### Immunocytochemistry (ICC)/immunohistochemistry (IHC)/microscopy

For ICC, tumor cells were plated on 1 mg/mL poly-l-lysine/laminin coated coverslips no. 1.0 or 1.5 (0.17 mm thick) and allowed to adhere overnight in DMEM-10. Cells were then rinsed twice in PBS, incubated for 15 min at room temperature in 4% paraformaldehyde, and then rinsed again three times in PBS. Cells were quenched with 50 mM ammonium chloride for 10 min and then permeabilized with 0.3% Triton X-100 in PBS (PBST) for 15 min. Following three PBS washes, coverslips were blocked with 5% normal serum for 1 h and then incubated overnight at 4 °C with target antibodies. The next day, cells were washed three times with PBS and then incubated with 1:2000 secondary antibody for 1 h; actin Green was added 30 min after the addition of the secondary antibody. Coverslips were then rinsed 3× in PBST prior to counterstaining with 0.04 μg/mL Hoechst (bisbenzimide). Followed three PBS washes, coverslips were mounted with Prolong Diamond (Invitrogen #P36971).

For IHC, we followed our previously published protocol^23^ with the following modifications: citrate buffer (pH 6.0 Vector #H-3300) was used for antigen retrieval in a steam cooker for 25 min and endogenous peroxidase activity was blocked for 10 min (Vector #SP-6000-100). For secondary antibody steps, Vector Labs’ DAB protocol was followed and HRP reactions were incubated for 3–4 min. Brightfield and fluorescent images were taken on an Olympus BX53 microscope with a DP80 dual CCD camera (CellSens imaging software) or z-stack images were acquired on a Zeiss AxioM2 using 0.13 µm × 0.13 µm × 1 µm sampling (MicroBrightField software). For confocal images, 3D cultured slides were imaged using a Zeiss LSM 880 NLO inverted laser scanning confocal microscope (Thornwood, NY) with a 63× 1.4 NA plan apochromat oil immersion objective in the Cell and Molecular Imaging Core of the Medical University of South Carolina. Hoechst (bisbenzimide) and phalloidin were excited at 405 nm and at 561 nm, respectively, and emission detected with a Quasar multichannel spectral detector at 410–665 nm (for DAPI) and at 566–689 nm (for phalloidin). Z-stacks were collected using 0.13 µm × 0.13 µm × 0.69 µm sampling and images were processed using Zen software (Zeiss).

### Live cell imaging

Cells were imaged on a Zeiss Observer.Z1 temperature, humidity and gas-controlled chamber with a 20× objective using Axiovision 4.7.1 software. Images were collected every 7 min for 24 h on both brightfield and fluorescence channels to capture overall cell morphology and trace fluorescently labeled nuclei.

### Transmission electron microscopy

Sub-confluent cells were fixed in 2% glutaraldehyde in 0.1 M cacodylate buffer for 30 min and rinsed overnight in cacodylate buffer with 7% sucrose. Cells were dehydrated through graded alcohols, infiltrated with a 1:1 solution of 100% ethyl alcohol and Embed 812 embedding resin for 30 min and embedded in Embed 812 embedding resin. Samples were polymerized for 24 h in a 60 °C oven and 24 h at room temperature. Thick sections were cut using a Riechert Ultramicrotome. Areas of interest were identified by light microscopy of 0.5-μm sections stained with toluidine blue. 70-nm thin sections were picked up on copper grids and double-stained with uranyl acetate and lead citrate prior to viewing on a JEOL 1210 transmission electron microscope.

### Determination of doubling times

2XSB cells were plated at densities of 10,000, 30,000 and 100,000 cells per well in a 24-well plate (Greiner; Monroe, NC) and then counted every 24 h out to 7 days post-plating. Duplicates were plated and counted on both a hemocytometer and a Celigo cytometer. All conditions and replicates were performed in triplicate.

### High density SNP microarray analyses

Genomic DNA was extracted from sporadic MPNST cells and the 2XSB parent tumor using a QIAamp Genomic DNA Purification Kit (Qiagen; Valencia, CA). Microarray-based chromosome analysis was performed with these DNAs using Infinium CytoSNP-850K BeadChips (Illumina, Inc.; San Diego, CA); these bead chips query 850,000 SNPs, with enriched coverage in 3262 dosage-sensitive genes. Copy number and genotype data was analyzed with KaryoStudio 1.2 (CNV Plugin V2.4.4.0; Illumina, Inc.), and Nexus 5.0 (BioDiscovery, Inc; El Segundo, CA) and interpreted using Human Genome Build 37/Hg19. Signal intensity, as assessed by the log_2_R ratio (LogR), and the specific allele (B allele) frequency was assessed to provide information regarding copy number and genotype, respectively. Deletions larger than 200 kb, duplications larger than 500 kb and regions of loss of heterozygosity greater than 3 Mb that were mosaic (not present in a percentage of cells less than 100%) were identified. Aberrations present in 100% of cells were considered constitutional and thus were not used for subsequent analyses.

### Whole exome sequencing

Genomic DNA was isolated using a QIAamp DNA Blood Mini Kit (Qiagen, Inc.; catalog number #51104) per the manufacturer’s recommendations. Genomic DNA was fragmented by sonication and then purified using Agencourt AMPure XP beads (Agencourt BioSciences Corporation; Beverly MA). Exome capture and library construction was performed using a SureSelectXT Human All Exon Kit (Agilent Technologies, Santa Clara CA), with index tags added by amplification of the captured exome. Whole exome paired-end sequencing (100 bp sequenced from each end) was performed using an Illumina HiSeq2000 instrument.

Sequence reads were aligned to human reference genome GRCm37/hg19 using DNASTAR SeqMan NGen software platform (version 14.0.0 build 88). Known and novel single nucleotide polymorphisms (SNPs), multiple nucleotide polymorphisms (MNPs) and small indels were identified using DNASTAR ArrayStar (version 14.0.0 build 85). QC metrics for cell line are: 133,022,154 total aligned reads, 97,195,438 total targeted aligned, 138 Mean region coverage depth, 95.74% uniformity of coverage; 99.92% aligned with reference genome. QC metrics for cell line are: 225,217,194 total aligned, 191,528,567 total targeted aligned, 222 mean region coverage depth, 66.93% uniformity of coverage, 98.77% aligned with reference genome. After global filtering to select for all variant calls that met our sequencing threshold (depth of coverage > 40 reads to be retained), the resulting variant calls were filtered through the variant effect predictor (VEP) to remove variants commonly encountered in the general population (variants with minor allele frequency greater than 0.01 in the 1000 Genomes Project dataset). To filter out benign SNPs from our target gene list, we assessed each of the variant calls using VarSome^80^, a combined automated scoring system that uses 18 criteria and data from 30 different databases. We then manually reviewed the evidence supporting VarSome’s interpretation that a non-synonymous variant was pathogenic or benign, paying particular attention as to whether the interpretation was supported by previous clinical evidence. The sequencing alignment and variant call reads from the final “pathogenic” and “likely pathogenic” gene list was then manually evaluated for a final QC check. In addition, we ran the FASTQ files through Varsome’s cancer variant caller platform as an additional confidence measure for variant calling. All reported variants were present in both platforms, had an allele depth of > 20 reads, a variant allele depth frequency of > 15% and displayed no strand bias. Fastq files were deposited to NCBI SRI database with BioSample accession numbers: SAMN17886490 and SAMN17886491.

### TERT promoter Sanger sequencing

TERT promoter sequences were amplified from tumor and cell line genomic DNA using primers targeting the region between − 270 and − 50 bps (underlined in primer sequence) upstream of the translational start site. Primers included upstream M13 sequencing tags: Forward primer: 5′TGT AAA ACG ACG GCC AGT GCC GGG CTC CCA GTG GAT TCG and Reverse primer: 5′CAG GAA ACA GCT ATG ACC GCT TCC CAC GTG CGC AGC AGG A. 50–100 ng of genomic DNA was amplified using 0.5 Units of Q5 High Fidelity Taq Polymerase (New England Biolabs), 0.5 μM of each primer, dNTPs, 1× Hot Start buffer and 1× Q5 High GC Enhancer under the following conditions: 1 cycle at 98 °C (3 min), followed by 42 cycles of 95 °C (15 s), 63 °C (15 s) and 72 °C (45 s); and then 1 cycle at 72 °C (5 min). PCR products were purified and used for Sanger sequencing. Primers and M13 facilitated sequencing were provided by Eurofins Genomics (Louisville, KY). Sequencing results were aligned to the TERT promoter and annotated in SnapGene, Version 5. Mutations were not called present unless they were identified in both forward and reverse sequencing reactions.

### Western blot analyses

Cells were homogenized in 1% SDS lysis buffer (100 mM NaCl and 1 mM Tris–Cl, pH 7.5.), boiled for 5 min and then centrifuged for 15 min in a microcentrifuge at full speed. Protein concentrations were determined using a Pierce BCA protein assay. Equal amounts of protein were resolved on 8% or 15% SDS–polyacrylamide gels, transferred to PVDF, blocked in 5% BSA in TBST (1 h at room temperature) and then probed with primary antibody overnight at 4 °C. The next day, blots were washed 3X in TBST and immunoreactivity was detected by incubating the blots with 1:20,000 Licor 680 or 800 fluorescent secondary antibodies in 5% BSA in TBST. Blots were imaged on a Licor instrument. Membranes were re-probed with an anti-actinin antibody (1:1000 dilution) as a loading control.

### Ras activation assays

Cells were grown to 70% confluency and then serum deprived in 0.05% serum containing growth media for approximately 16 h. After stimulating cells with 10% serum for 5 min, lysates were isolated from treated cells and immediately prepped for pull-down experiments using 0.5–1 mg of lysate per the manufacture’s protocol (Millipore #17-218). Pull-down material was resolved by PAGE on a 4–15% gradient gel followed by immunoblot analysis. All experiments were performed in triplicate.

### Xenografts

2 × 10^6^ 2XSB cells in 50% Matrigel were injected subcutaneously into 6 to 10 week old NOD-Scidγ mice. Animals were monitored several times weekly. Just prior to grafts reaching the maximum size allowed by IACUC (< 1 cm), the mice were euthanized, and tissue was harvested. A portion of the graft was flash frozen, with the remainder being fixed in 4% paraformaldehyde overnight and then processed for paraffin embedding.

## Supplementary Information

Supplementary Video 1.

Supplementary Video 2.

Supplementary Video 3.

Supplementary Table 1.

Supplementary Table 2.

Supplementary Table 3.

Supplementary Table 4.

Supplementary Figures.

## Data Availability

TheFastq files for the 2XSB cell line and parent tumor WES datasets reported in this manuscript have been deposited in the NCBI SRI database with BioSample accession numbers SAMN17886490 and SAMN17886491, respectively, and will be released upon paper acceptance.
