# Supplementary material for: Establishment and genomic characterization of a sporadic malignant peripheral nerve sheath tumor cell line
Source: Sci Rep. 2021 Mar 11;11:5690. doi: 10.1038/s41598-021-85055-2 (PMC7952412; doi:10.1038/s41598-021-85055-2)

Supplemental Materials

Establishment and genomic characterization of a sporadic malignant peripheral nerve sheath tumor cell line

Jody Fromm Longo[1], Stephanie N. Brosius[3,5*], Iya Znoyko[1], Victoria A. Alers[1], Dorea P. Jenkins[1], Robert C. Wilson[1,2], Andrew J. Carroll[4], Daynna J. Wolff[1], Kevin A. Roth[6] and Steven L. Carroll[1,2,3**]

Departments of Pathology and Laboratory Medicine[1] and the Center for Genomic Medicine[2], Medical University of South Carolina, Charleston, SC 29425-9080
Departments of Pathology[3] and Genetics[4] and the Medical Scientist Training Program[5], University of Alabama at Birmingham, Birmingham, AL 35294-0017
Department of Pathology and Cell Biology[6], Vagelos College of Physicians and Surgeons, Columbia University, New York, NY 10032

Supplemental Figure S1

RNA Sequencing analysis

Name	/gene_synonym	2XSB RPKM	Tumor RPKM
MLANA	MART-1, MART1	0.251	0.07
PMEL	D12S53E, gp100, ME20, ME20-M, ME20M, P1, P100, PMEL17, SI, SIL, SILV	0.18	0.653
TERT	CMM9, DKCA2, DKCB4, EST2, hEST2, hTRT, PFBMFT1, TCS1, TP2, TRT	0.197	0.125

Supplemental Figure S2

3D Culture of Giant only cells

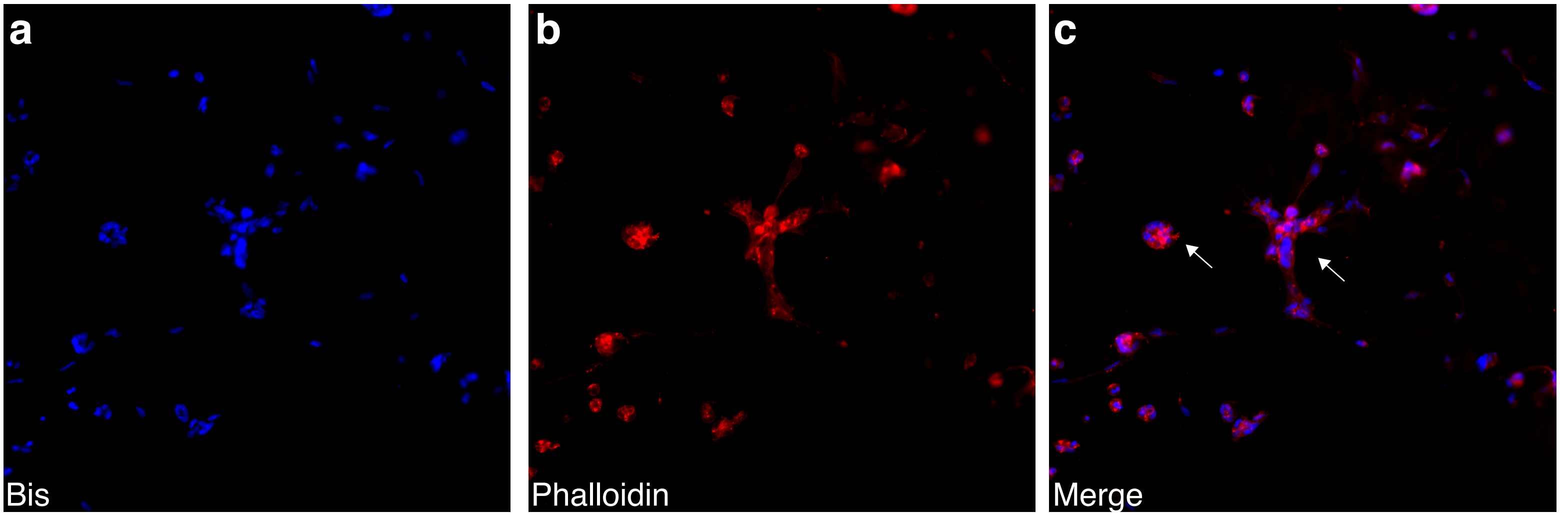

d Xenograft Growth Measurements

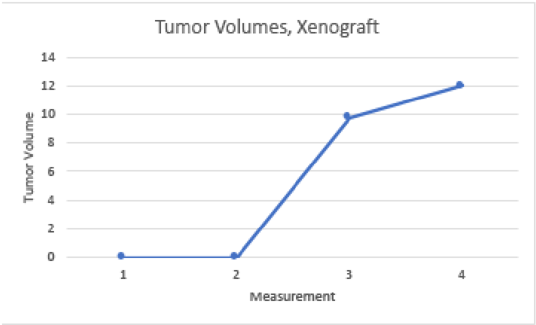

Supplemental Figure S3

a Tumor

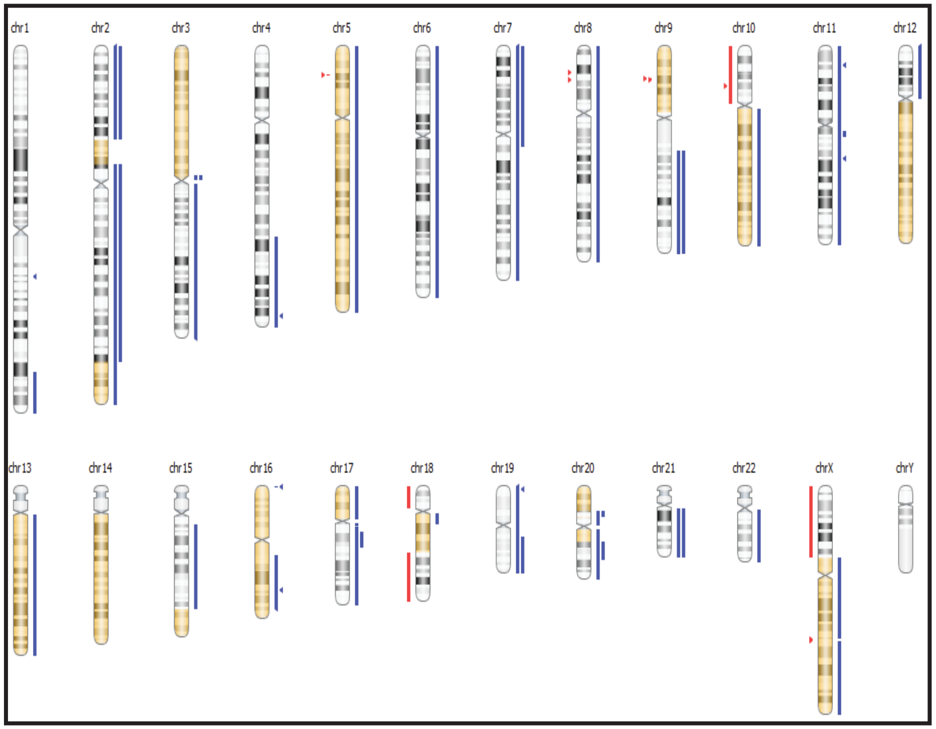

b Cell Line

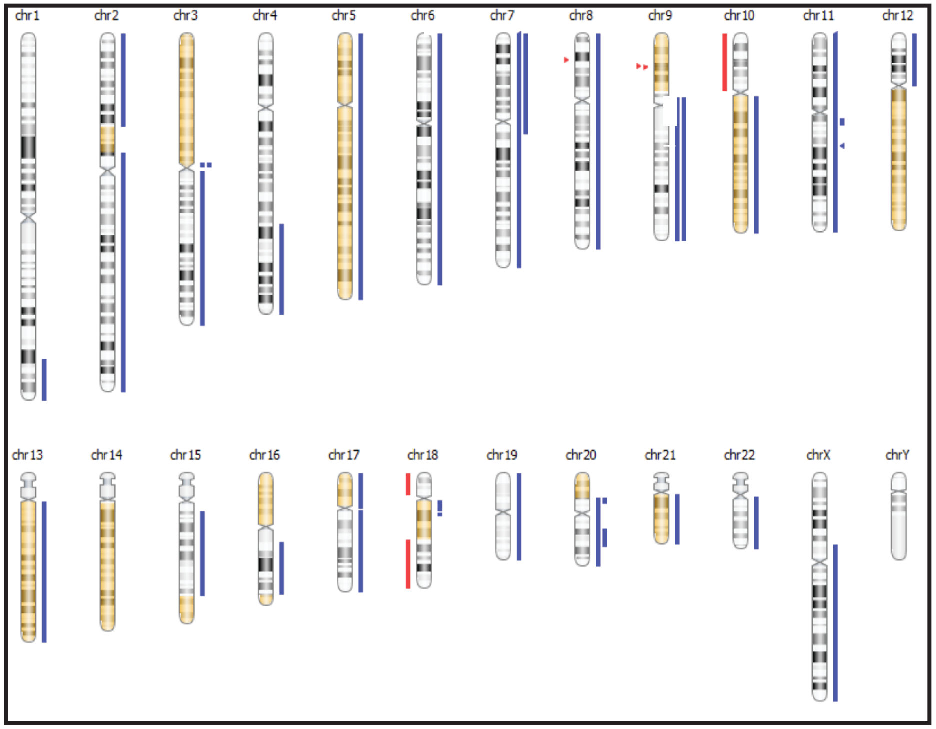

Supplemental Figure S4

a Tumor

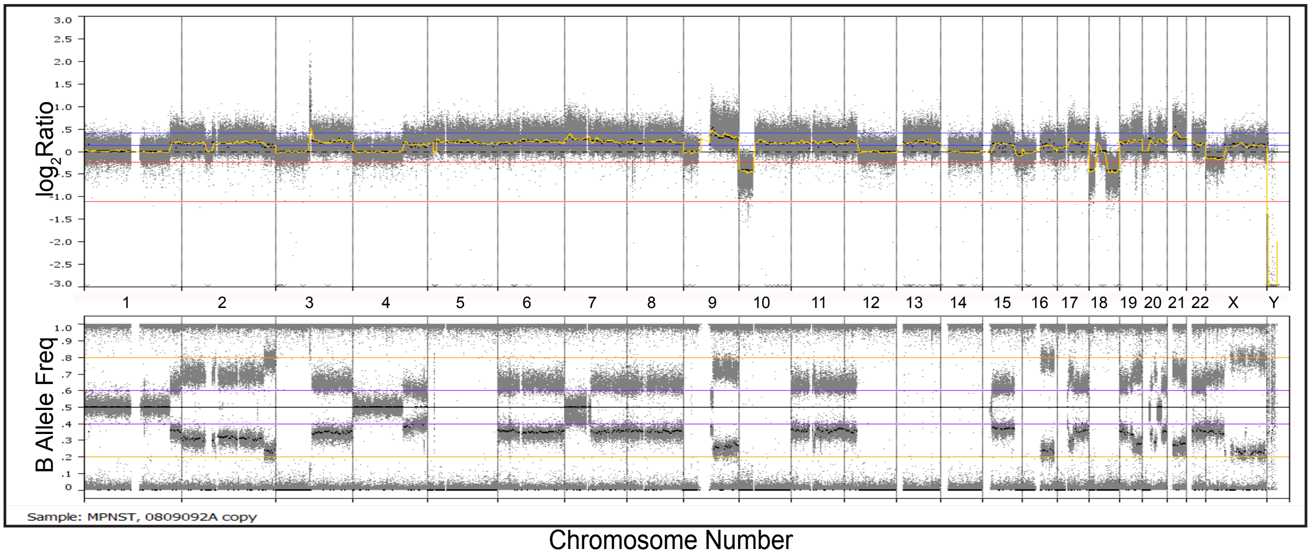

b Cell Line

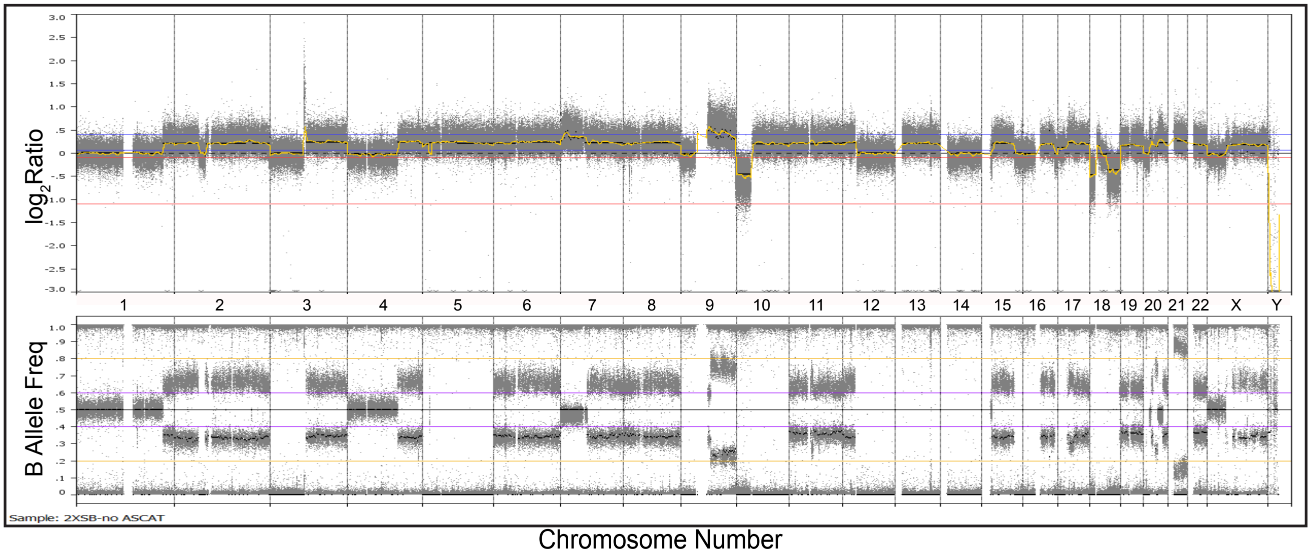

Supplemental Figure S5

a **NF1 (tumor)**

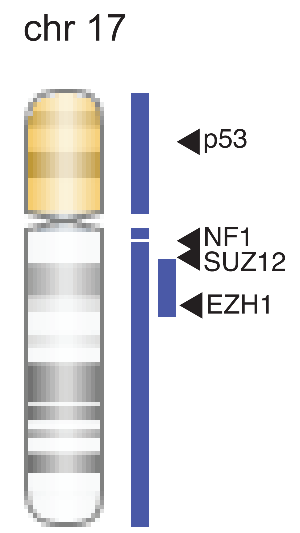

b **CDKN2A (tumor)**

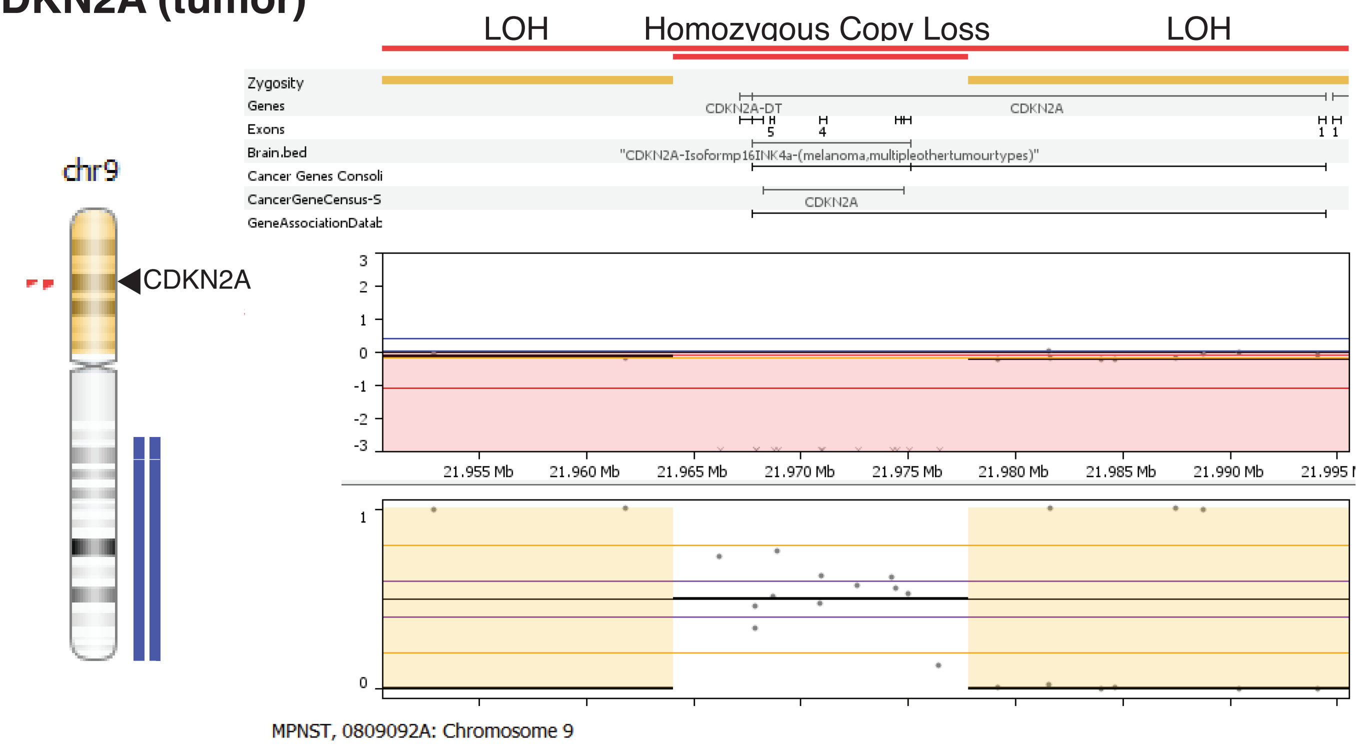

Supplemental Figure S6

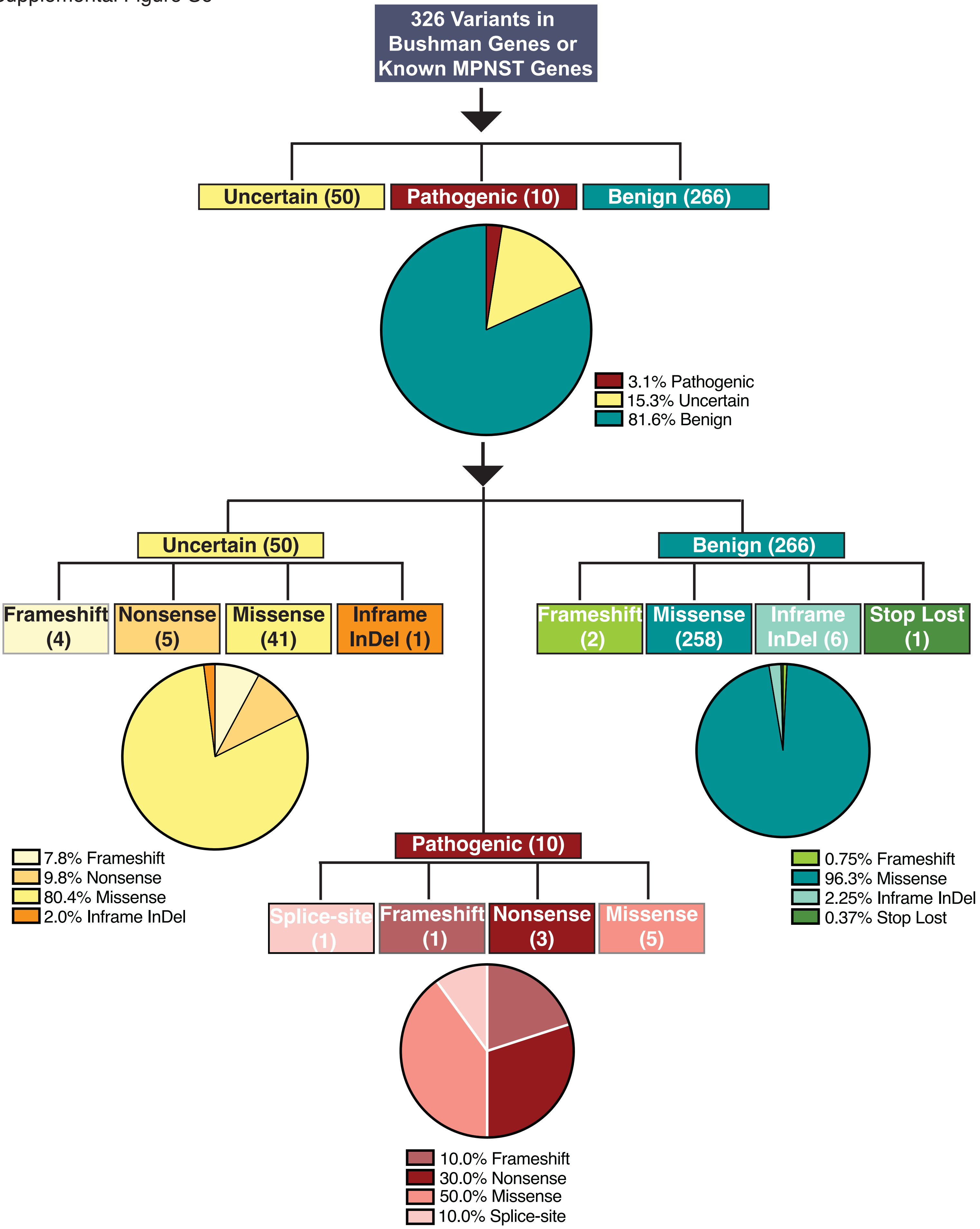

Supplemental Figure S7

WES assembly output: TP53, p.H178D

Tumor:

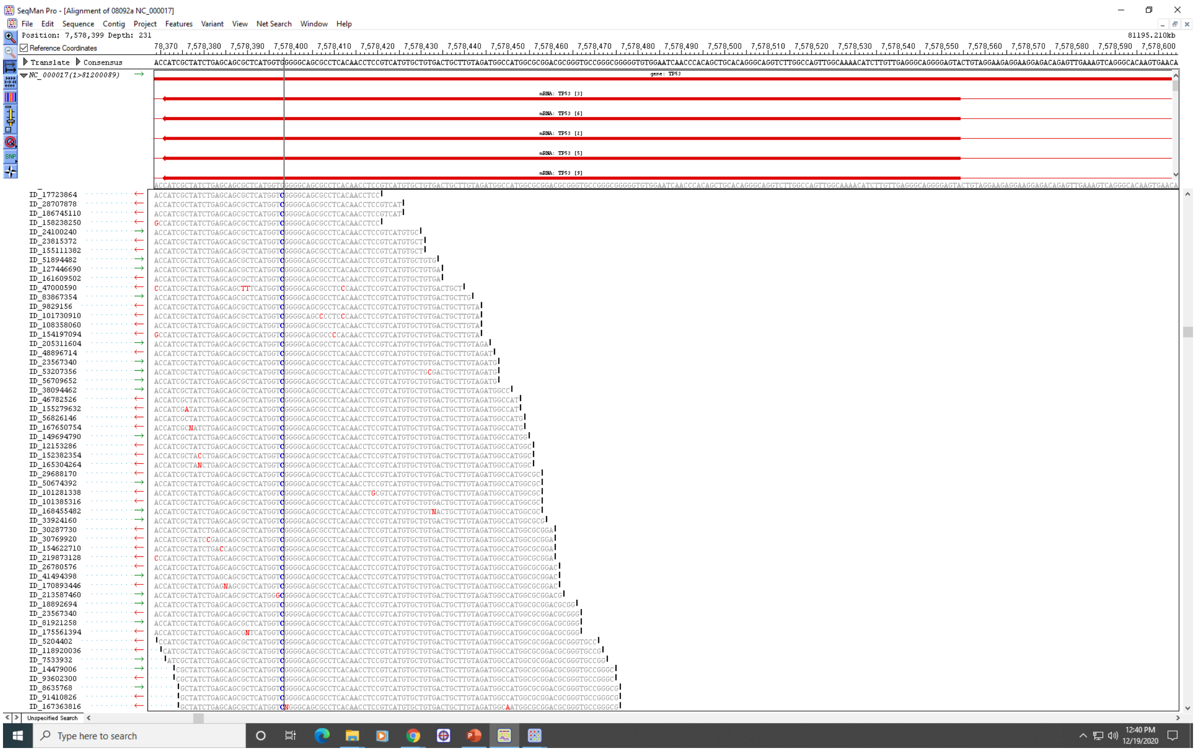

Cell line:

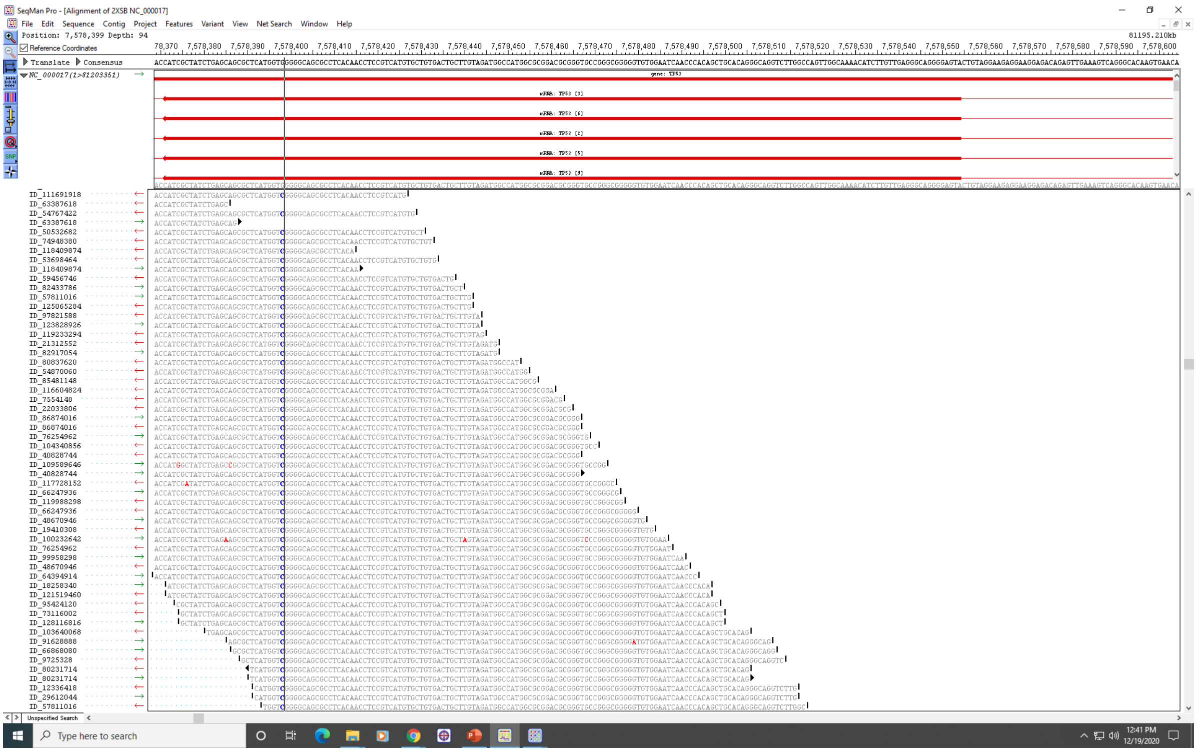

Supplemental Figure S7

WES assembly output: PTEN, p.P38S

Tumor:

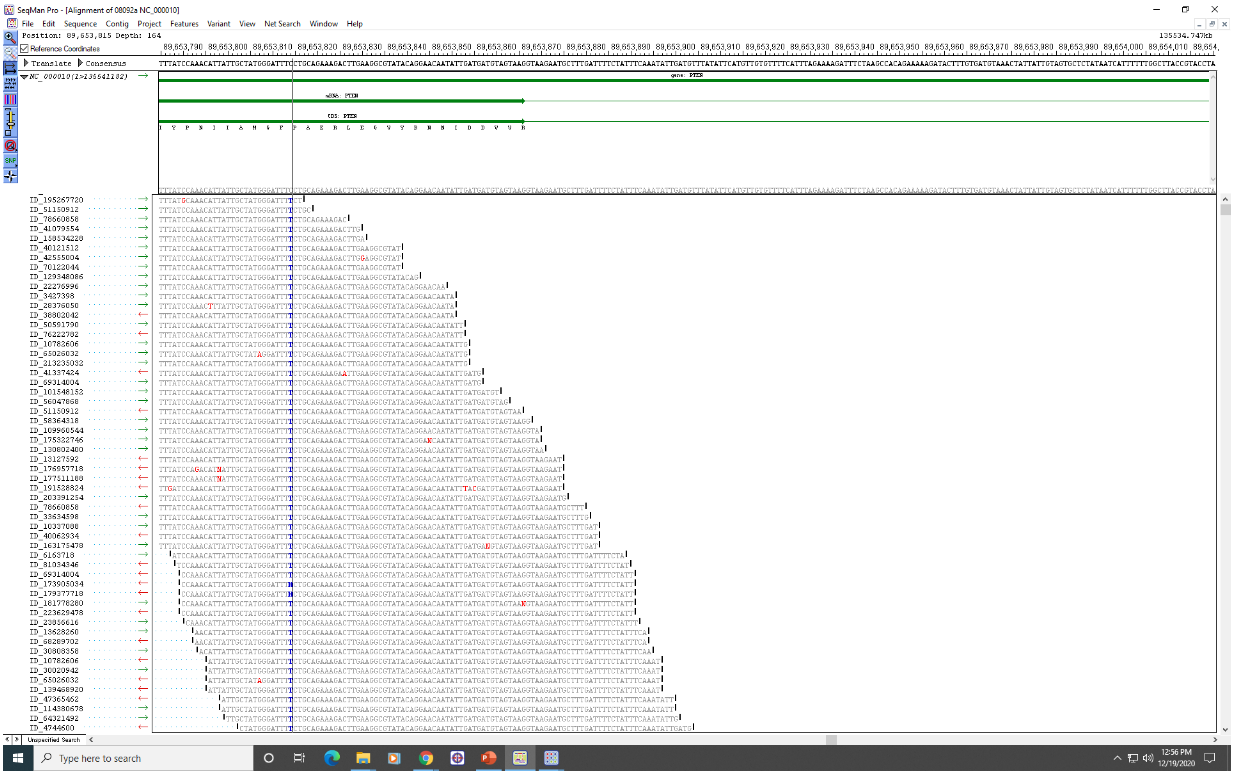

Cell line:

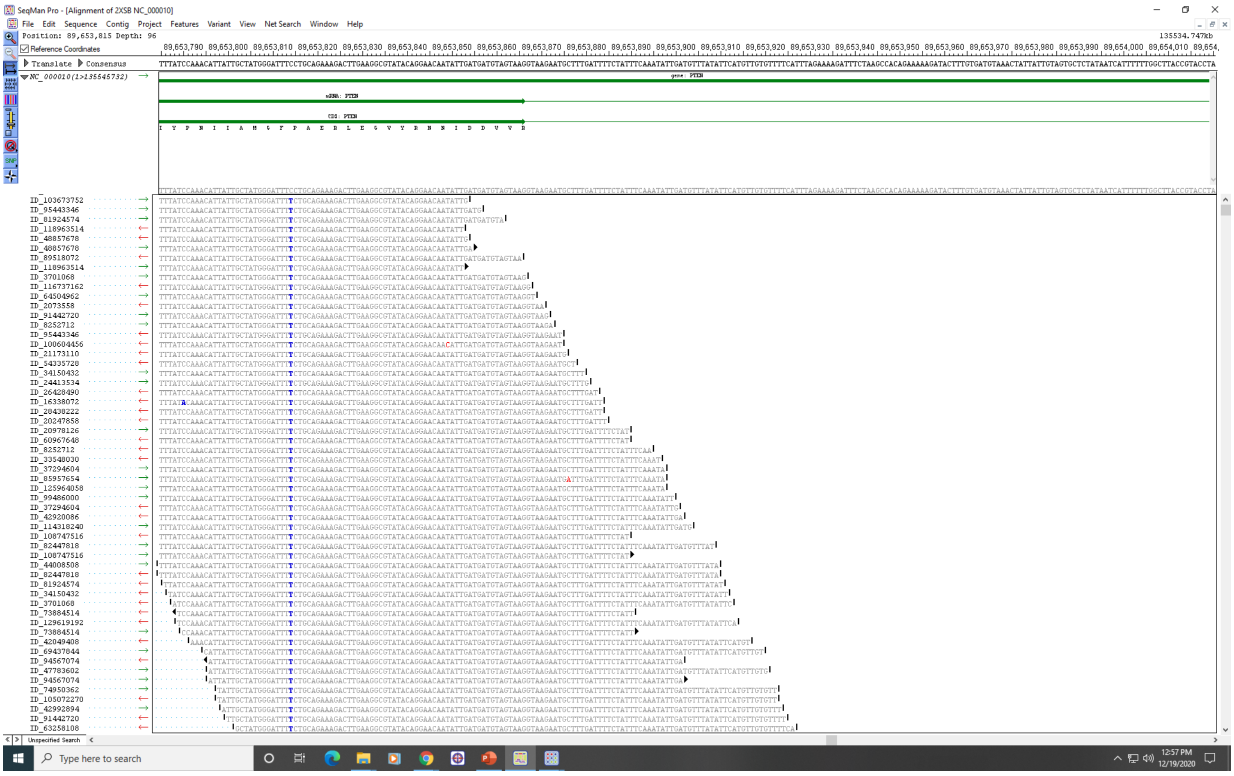

Supplemental Figure S7

WES assembly output: DNMT1, c.2586+1 G>A

Tumor:

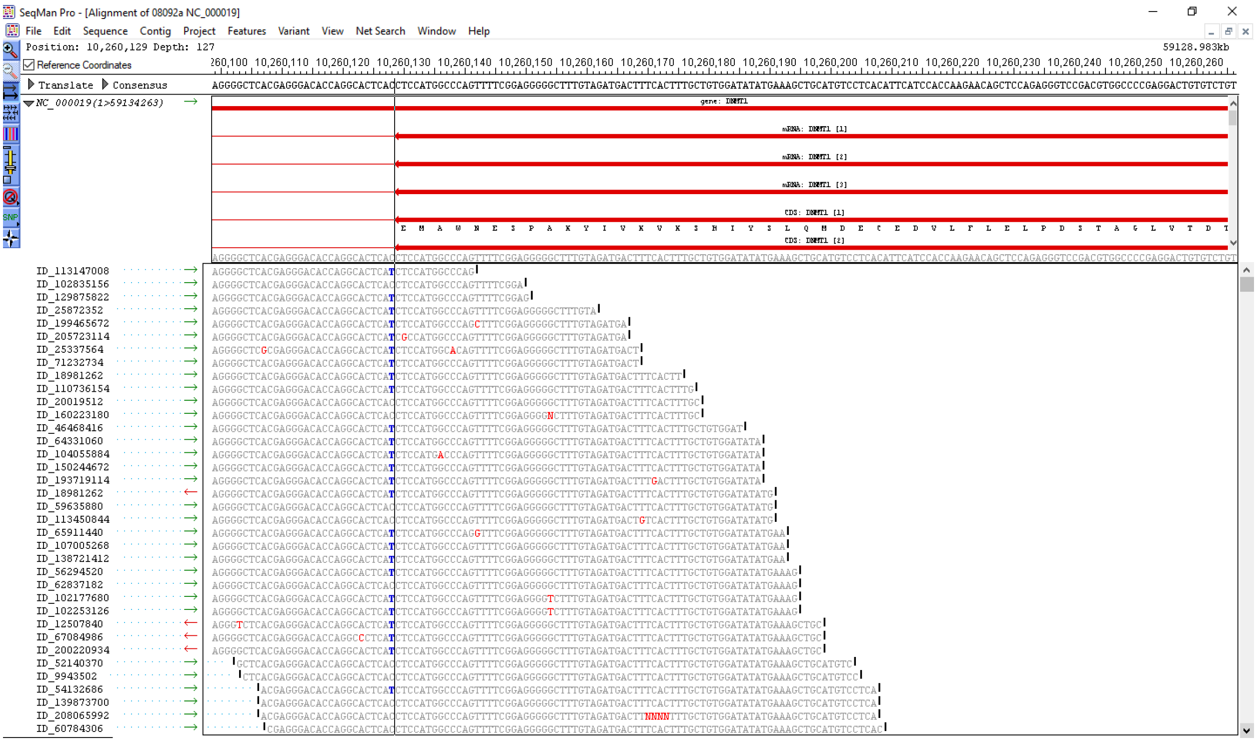

Cell line:

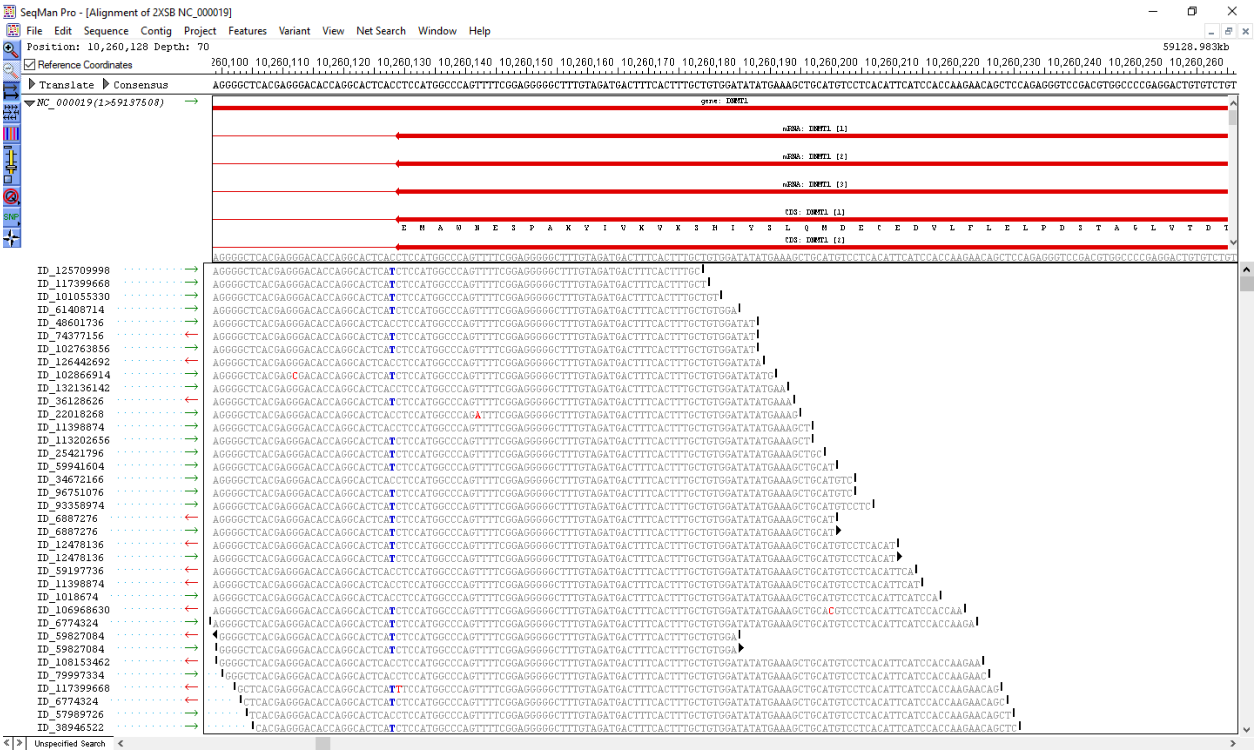

Supplemental Figure S7

WES assembly output: NUMA1, p.L1167fs

Tumor:

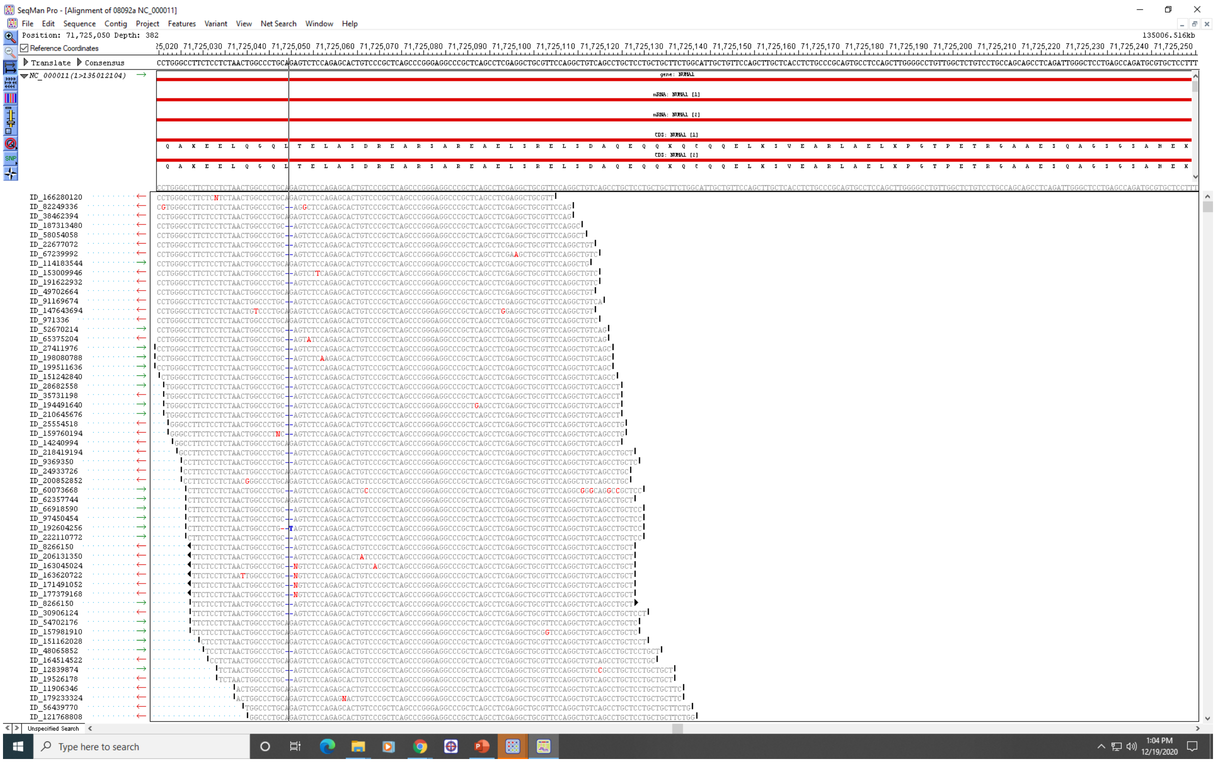

Cell line:

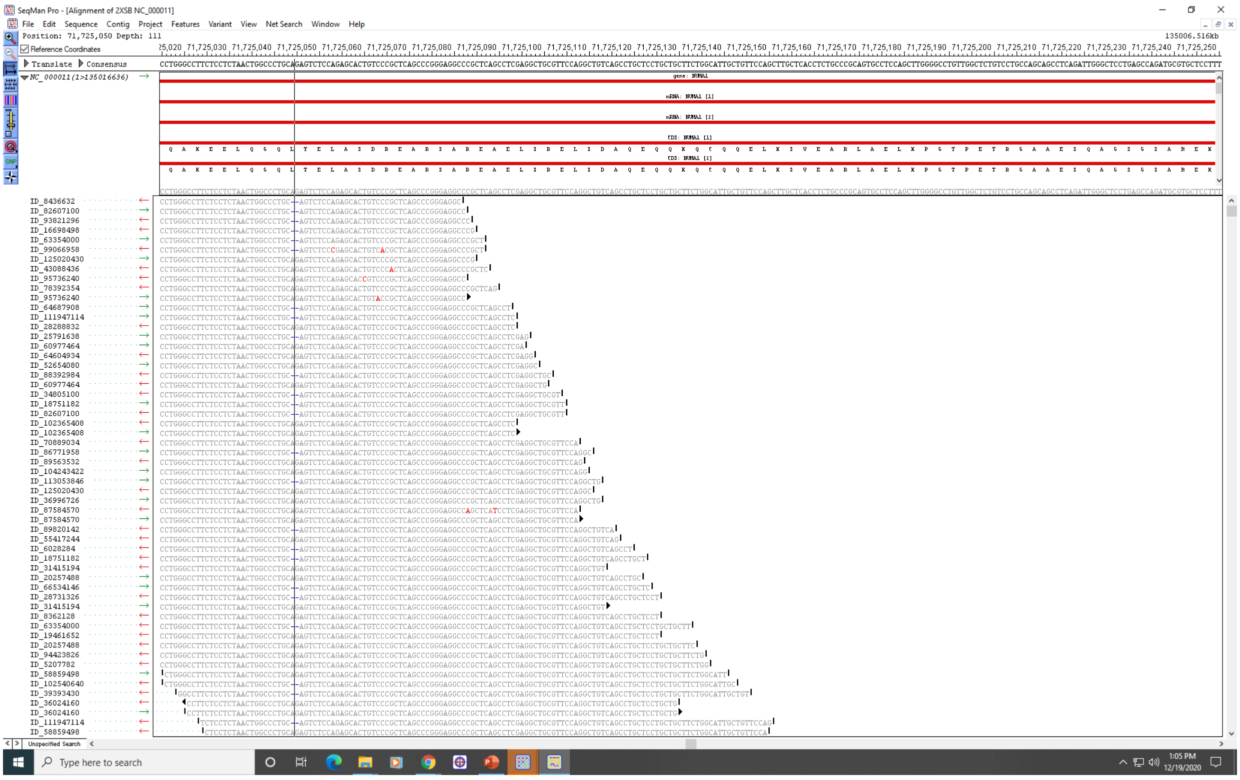

Supplemental Figure S7

WES assembly output: NTRK1, p.R677C

Tumor:

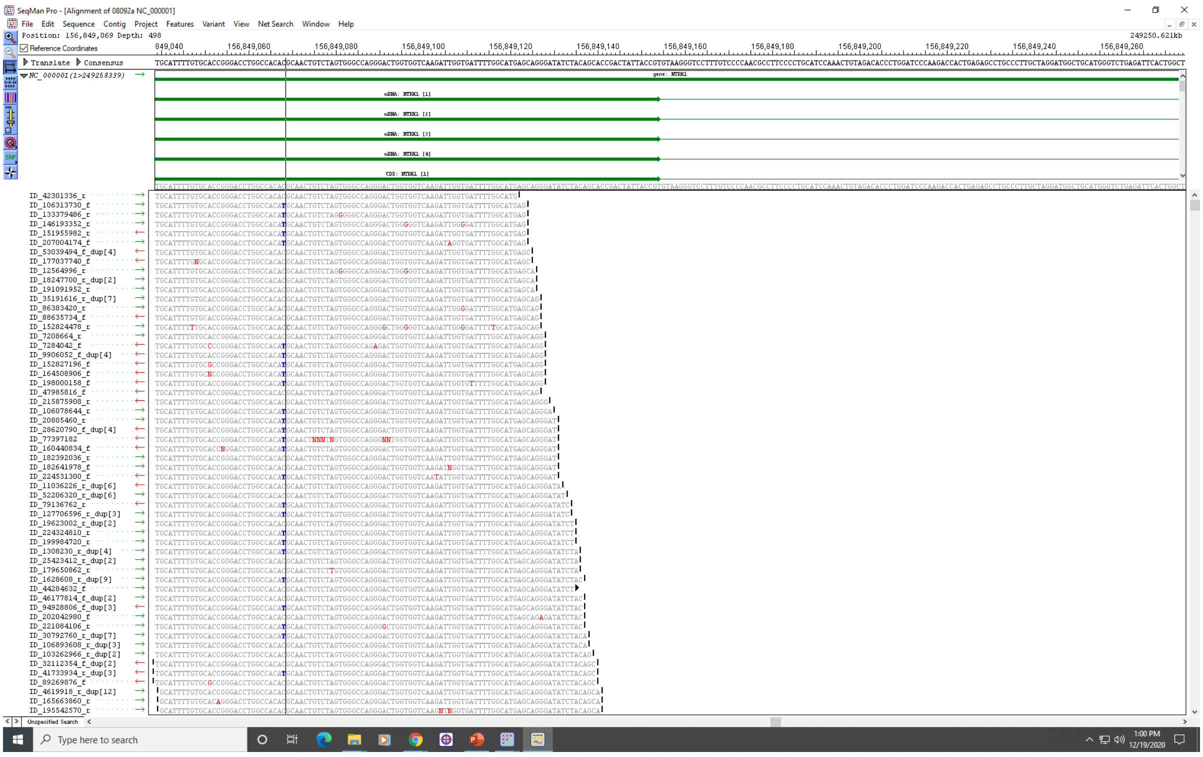

Cell line:

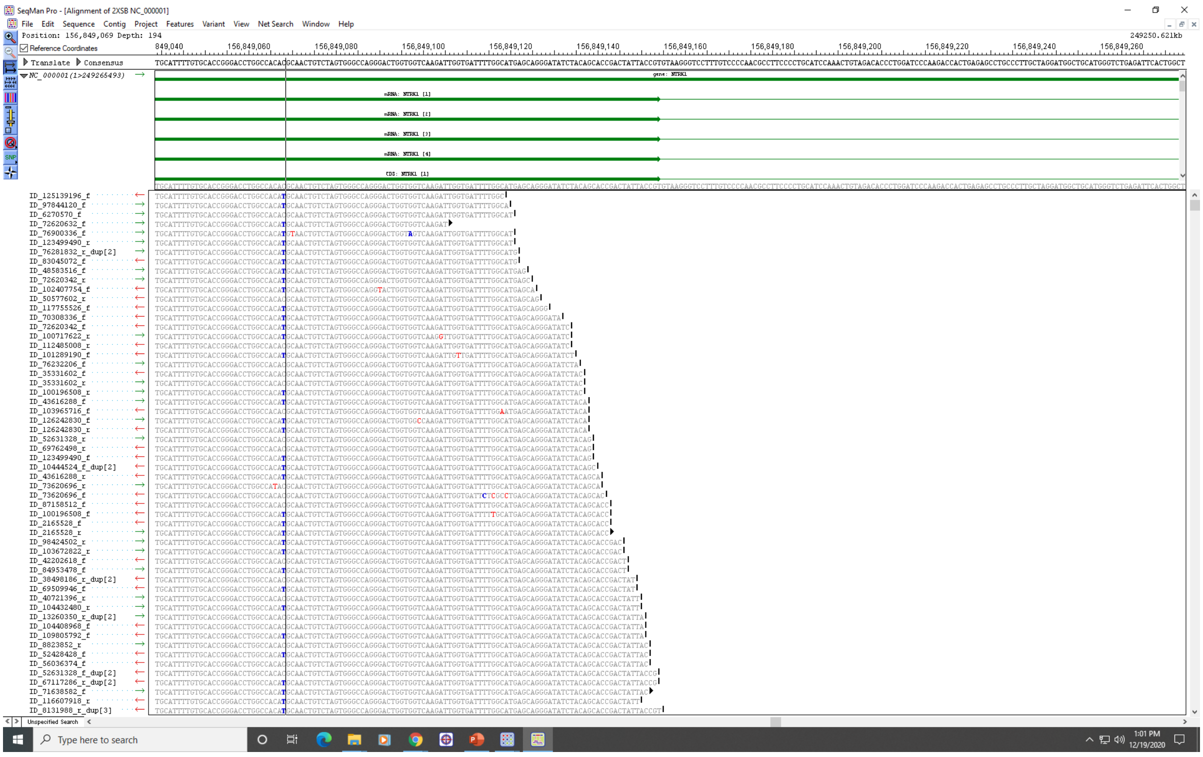

Supplemental Figure S7

WES assembly output: PDE11A, p.P434L

Tumor:

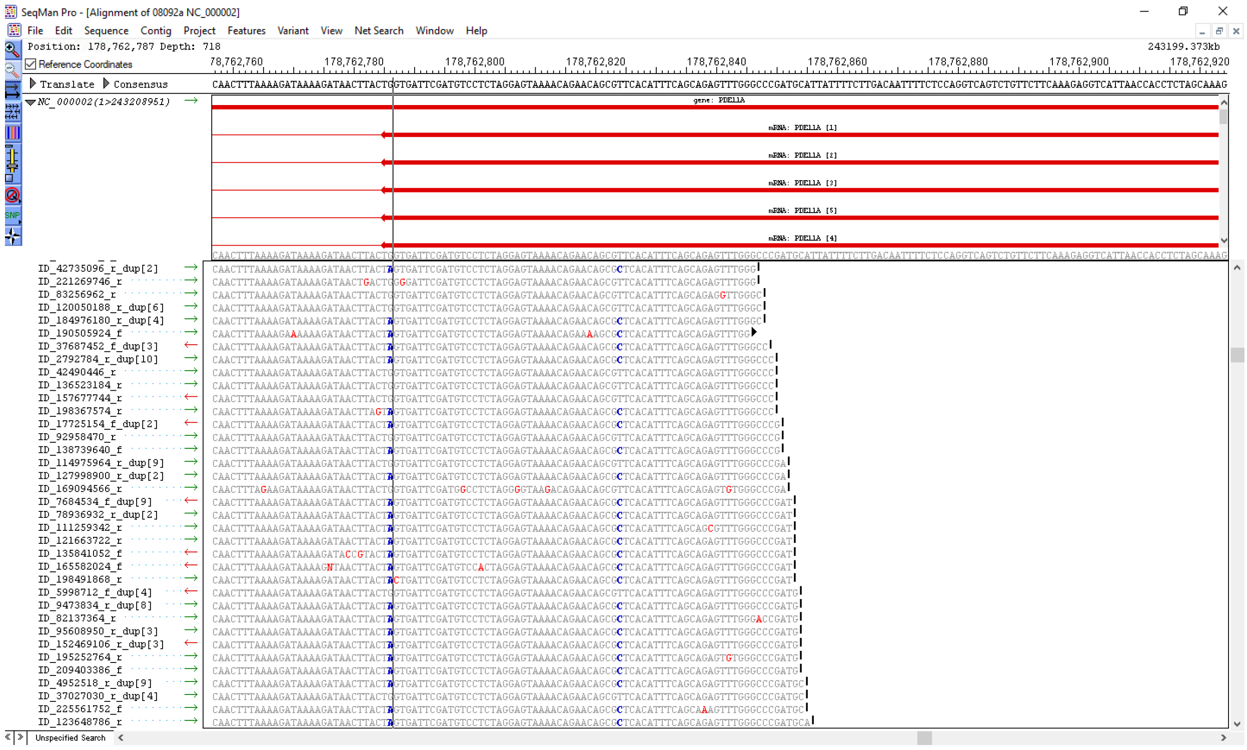

Cell line:

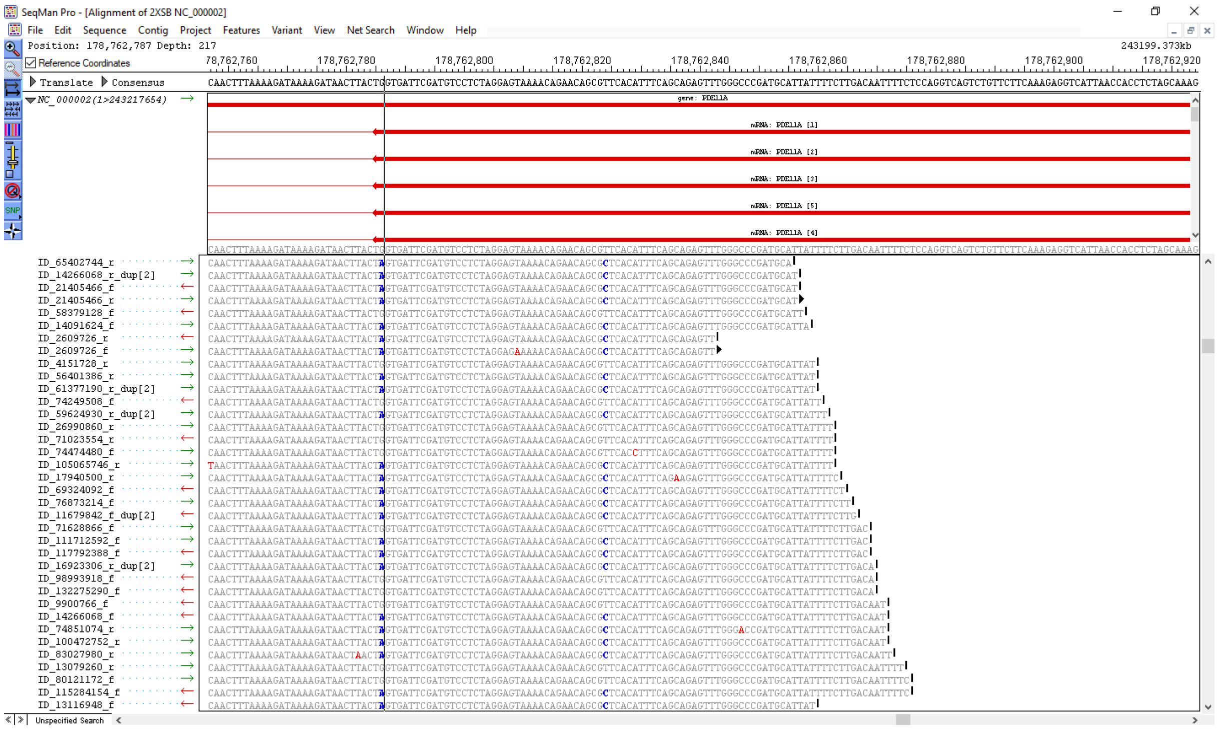

Supplemental Figure S7

WES assembly output: BRAF, p.V600E

Tumor:

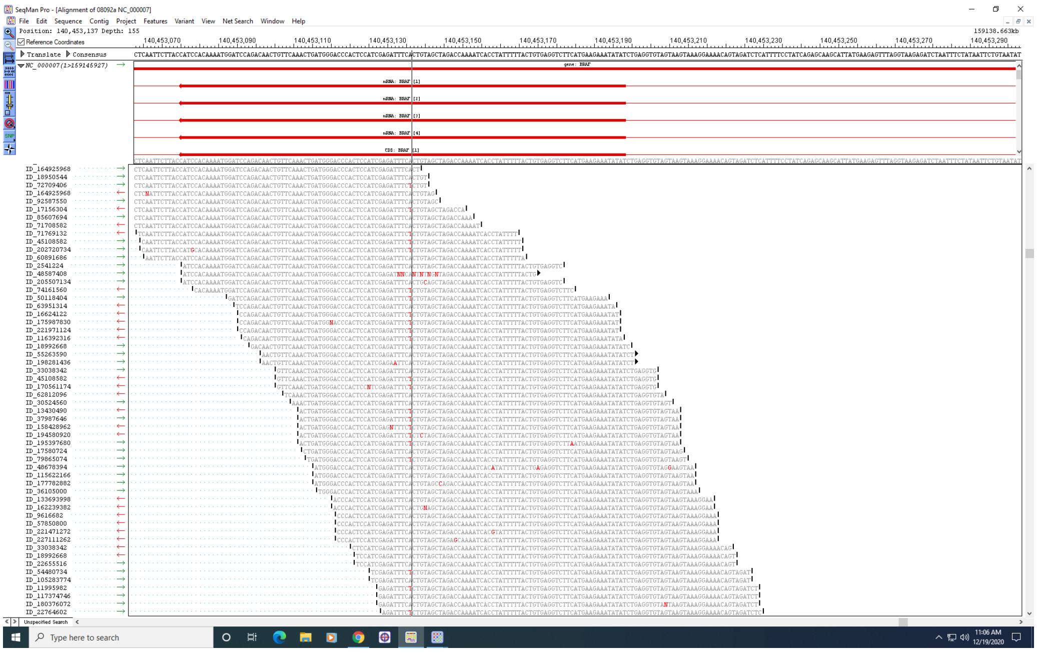

Cell line:

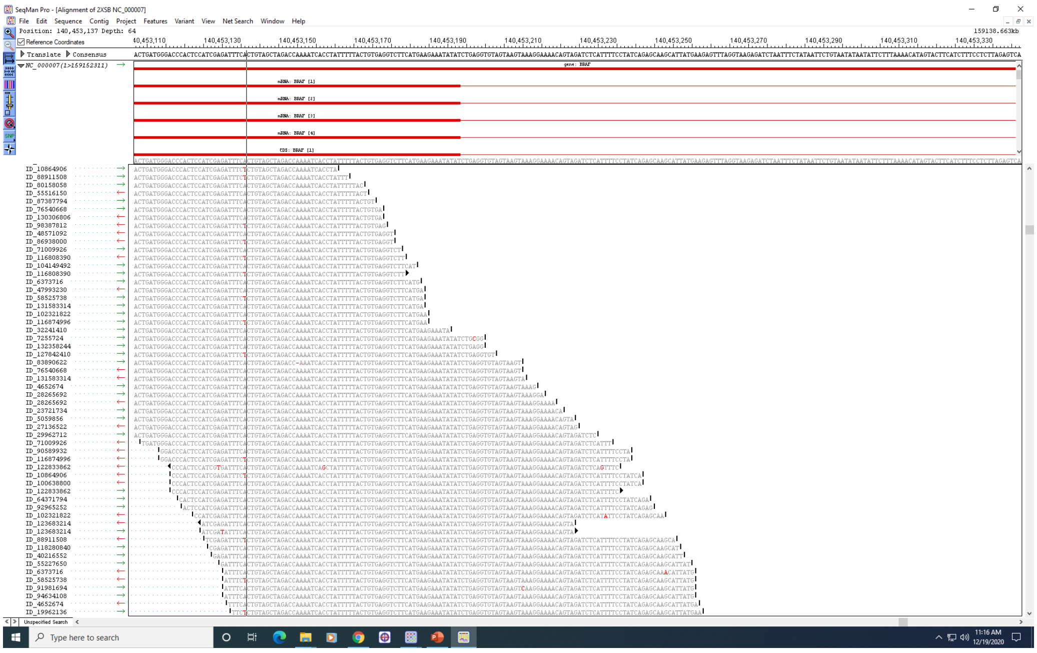

Supplemental Figure S7

WES assembly output: CSMD3, p.W3337*

Tumor:

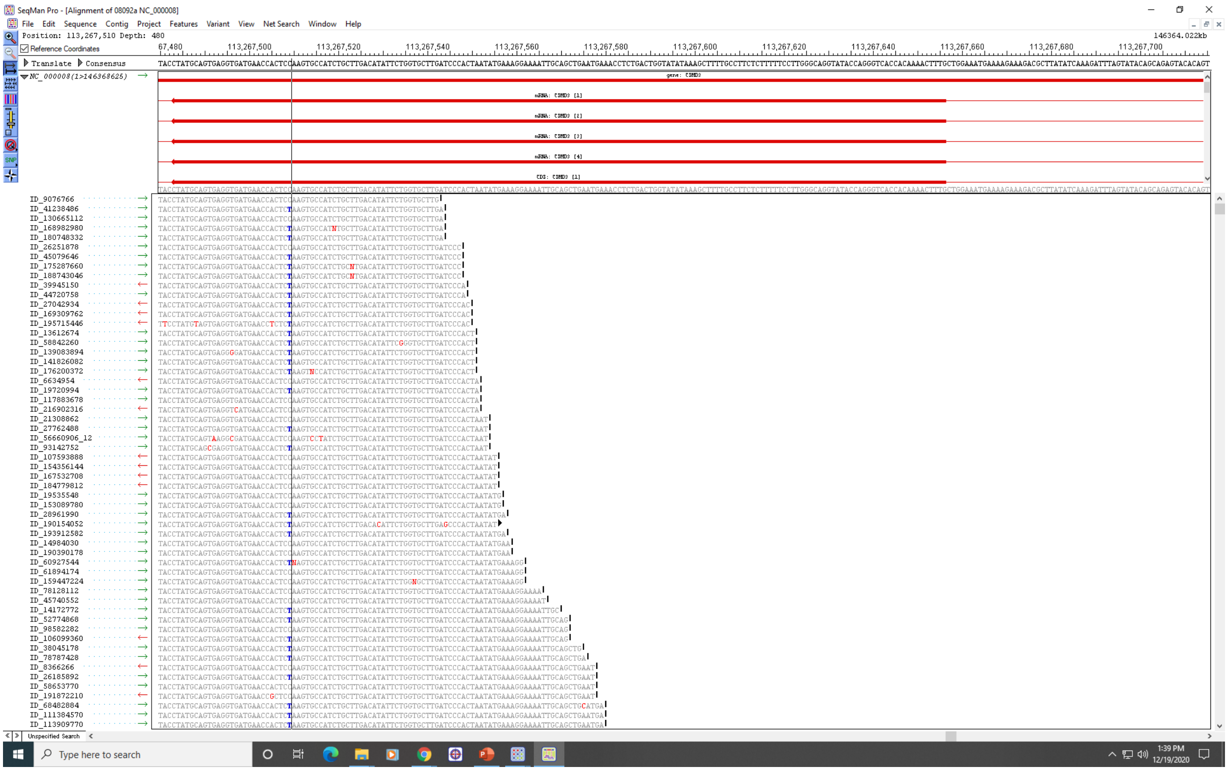

Cell line:

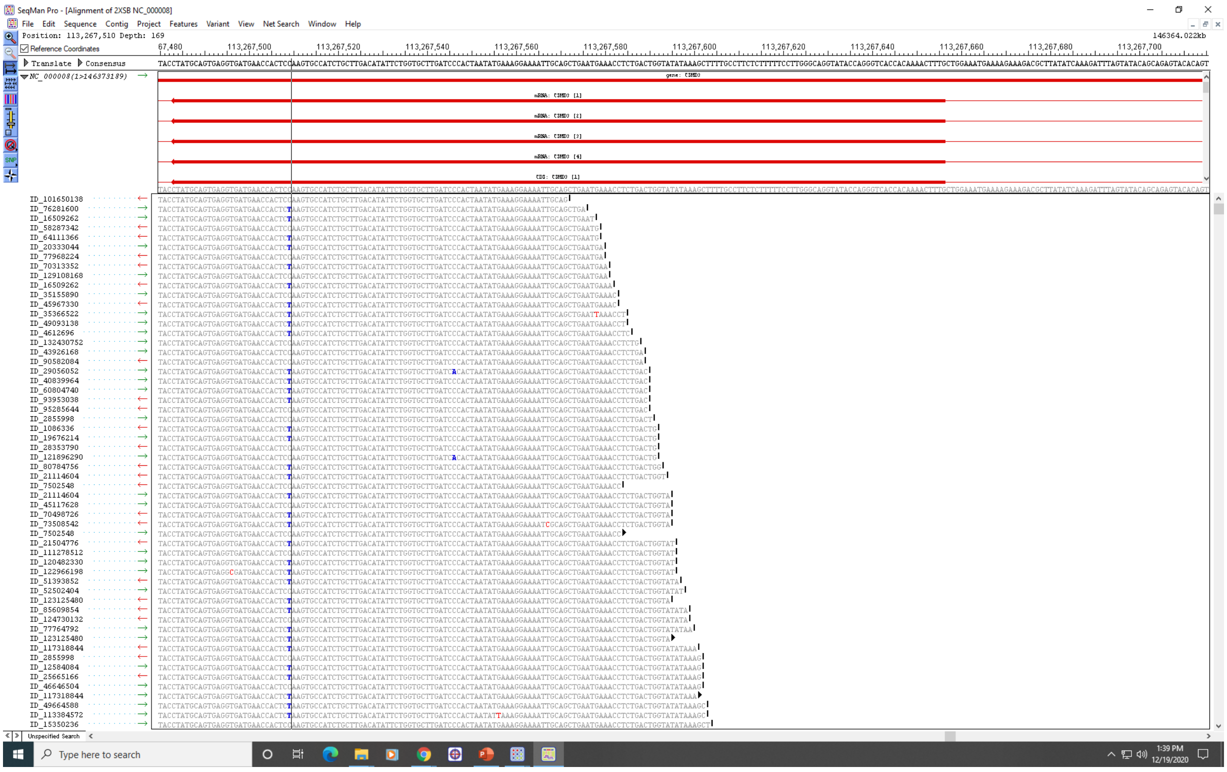

Supplemental Figure S7

WES assembly output: LRP5, p.R428*

Tumor:

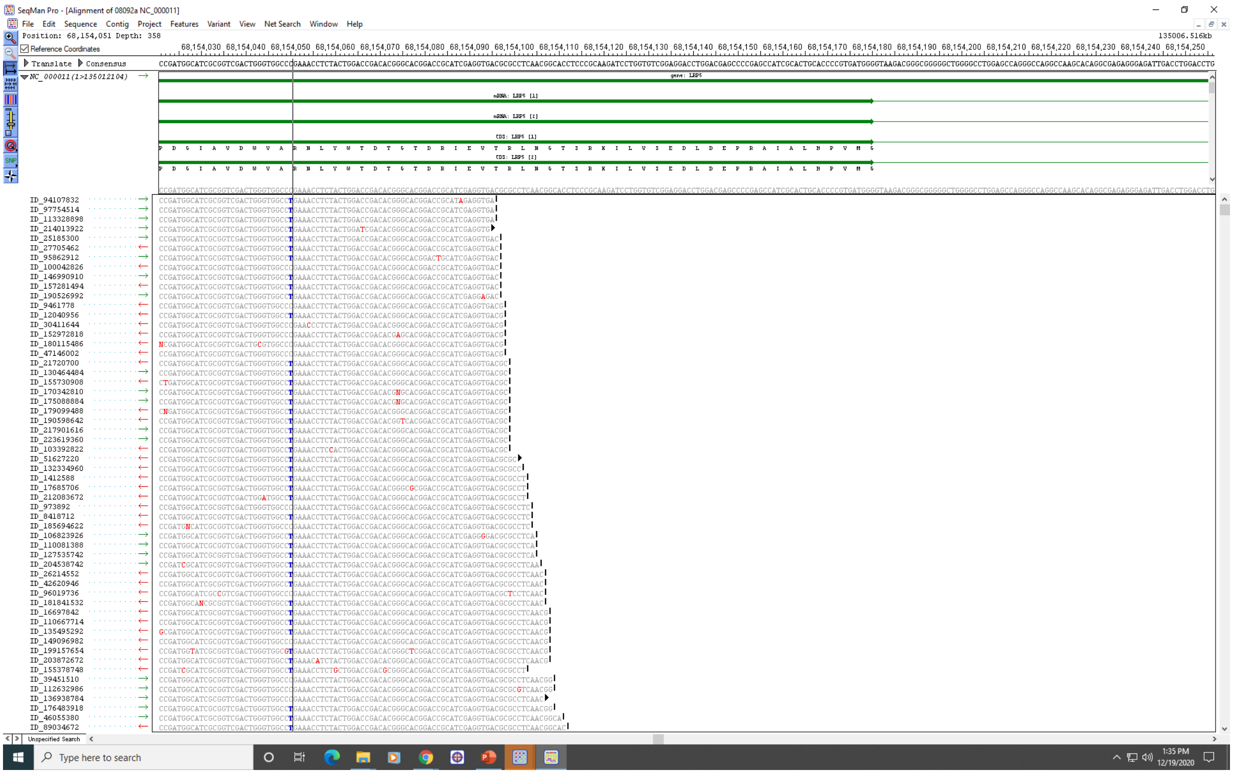

Cell line:

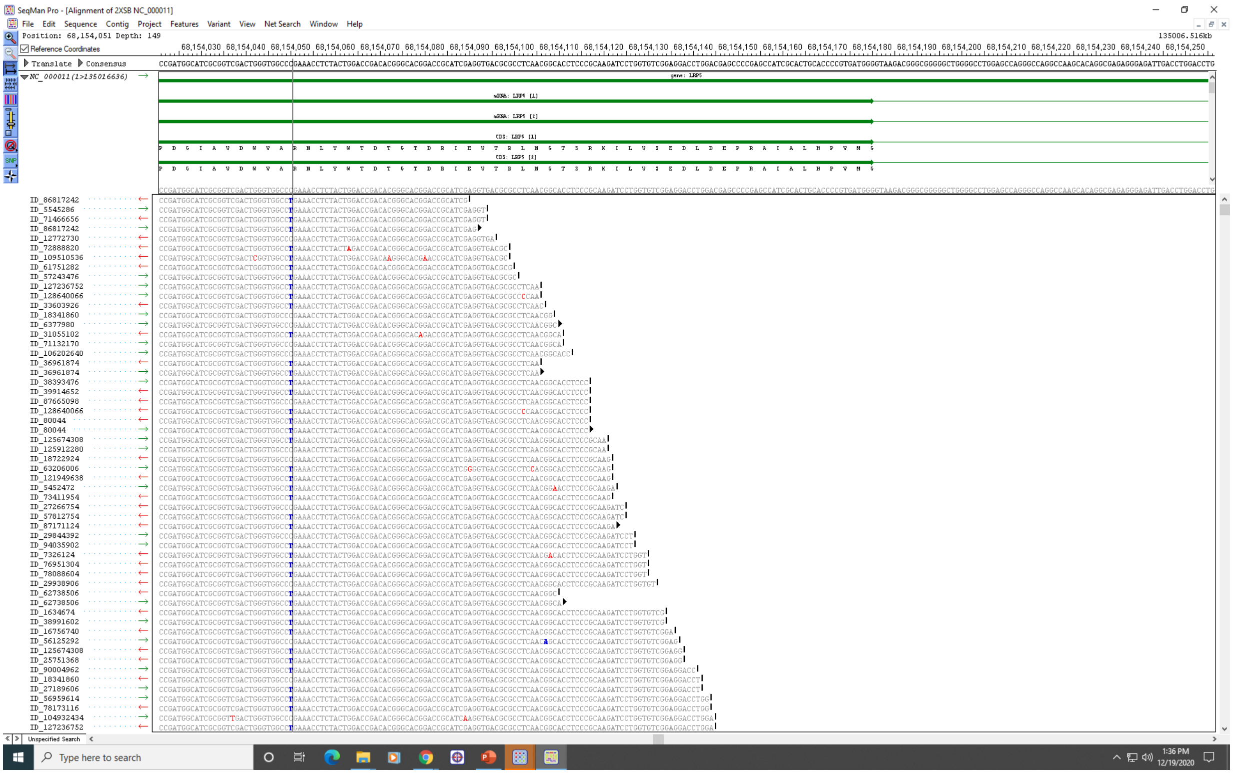

Supplemental Figure S7

WES assembly output: ACTL9, p.Q12*

Tumor:

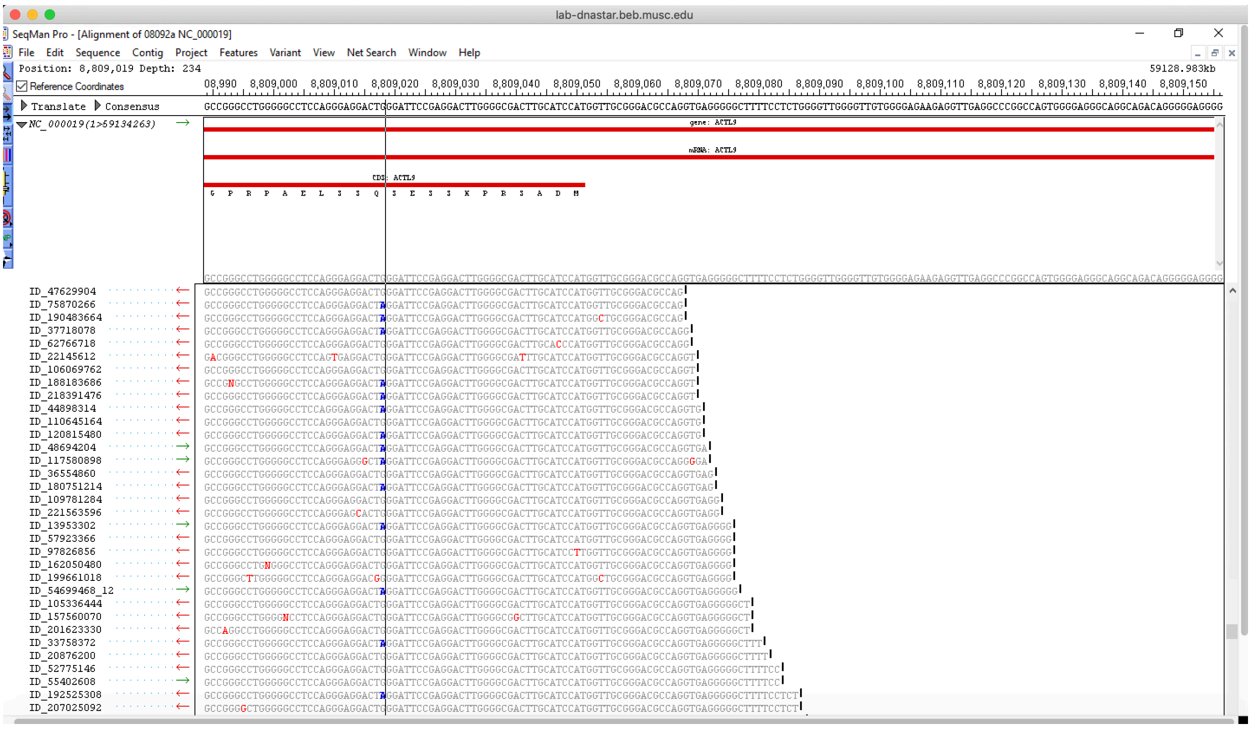

Cell line:

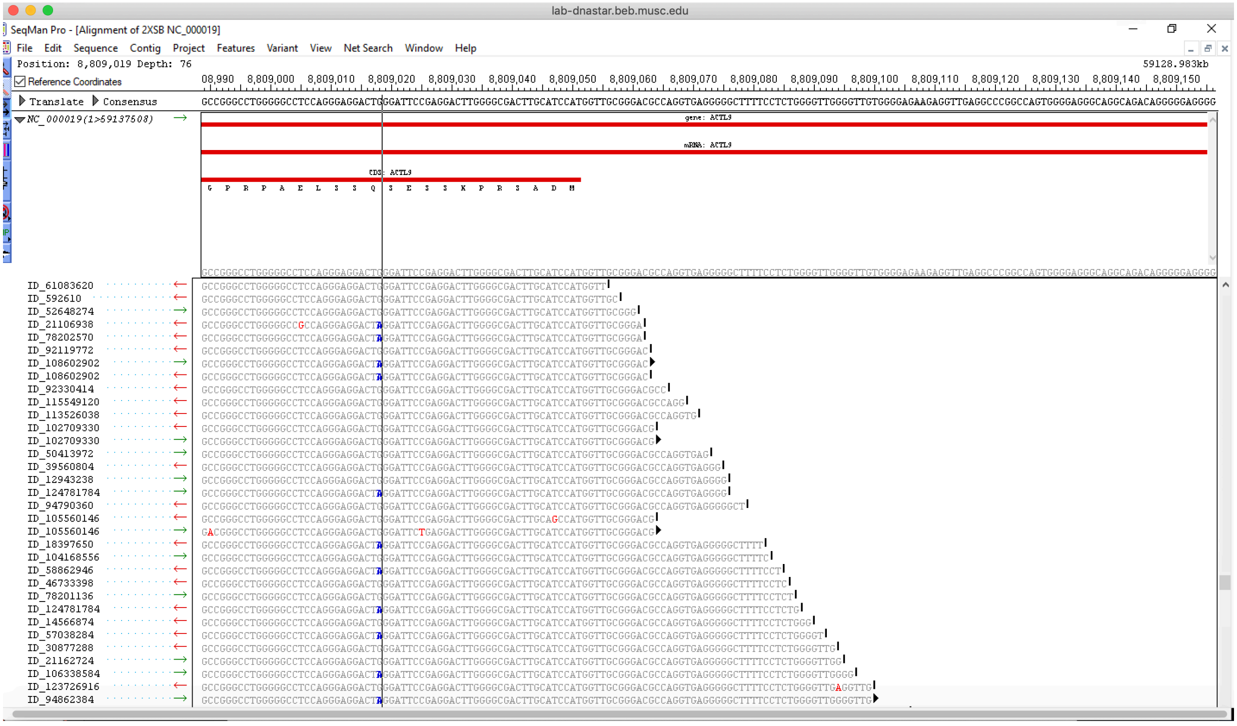

Supplemental Figure S8a

Sanger Sequencing TERT Promoter Mutational Analysis, Schematic

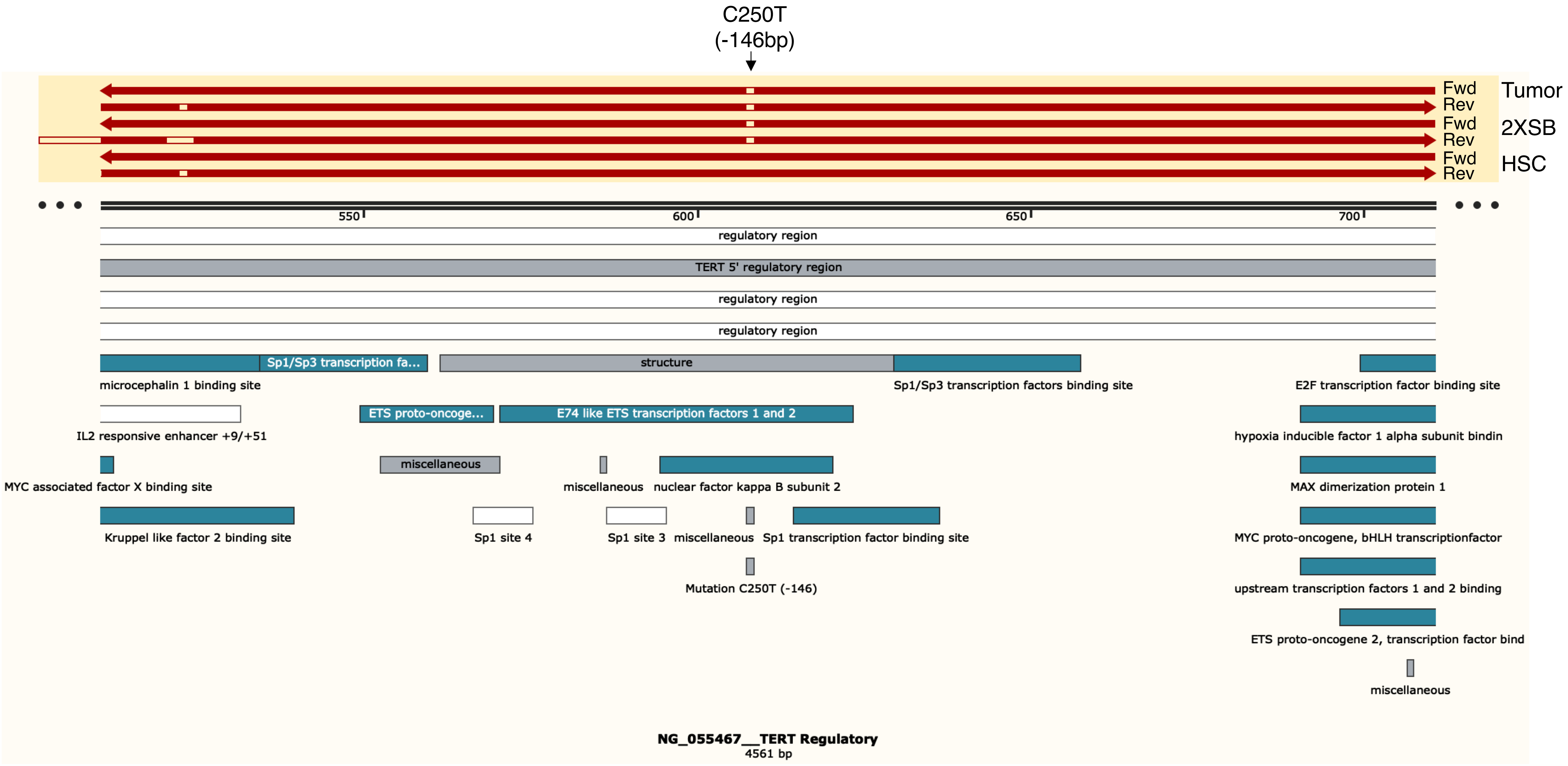

Supplemental Figure S8b

Sanger Sequencing TERT Promoter Mutational Analysis, Sequence

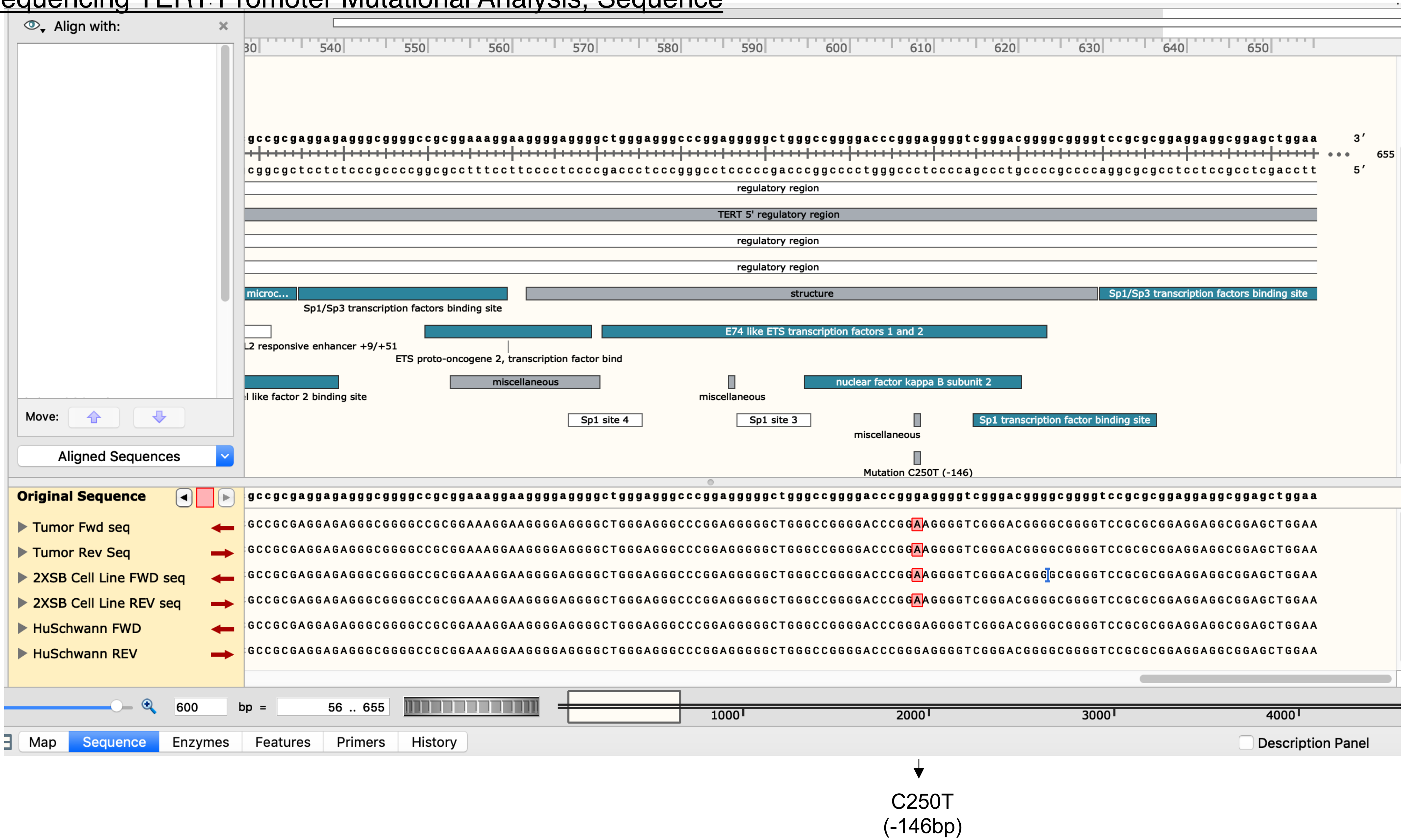

Supplemental Figure S8c

Sanger Sequencing TERT Promoter Mutational Analysis, Other MPNST Lines, Schematic

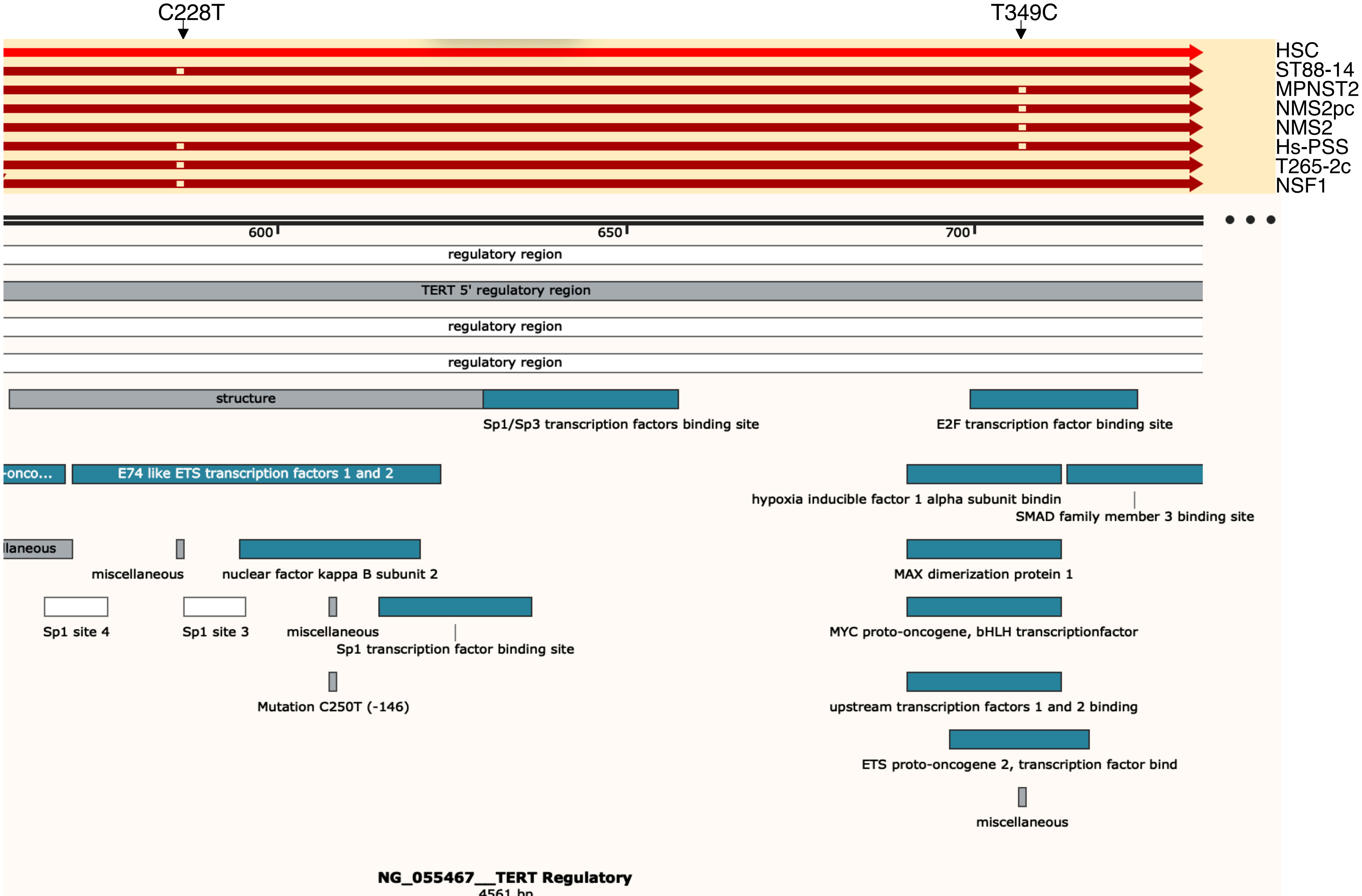

Supplemental Figure S8d

<u>Sanger Sequencing TERT Promoter Mutational Analysis, Other MPNST Lines, Sequence</u>

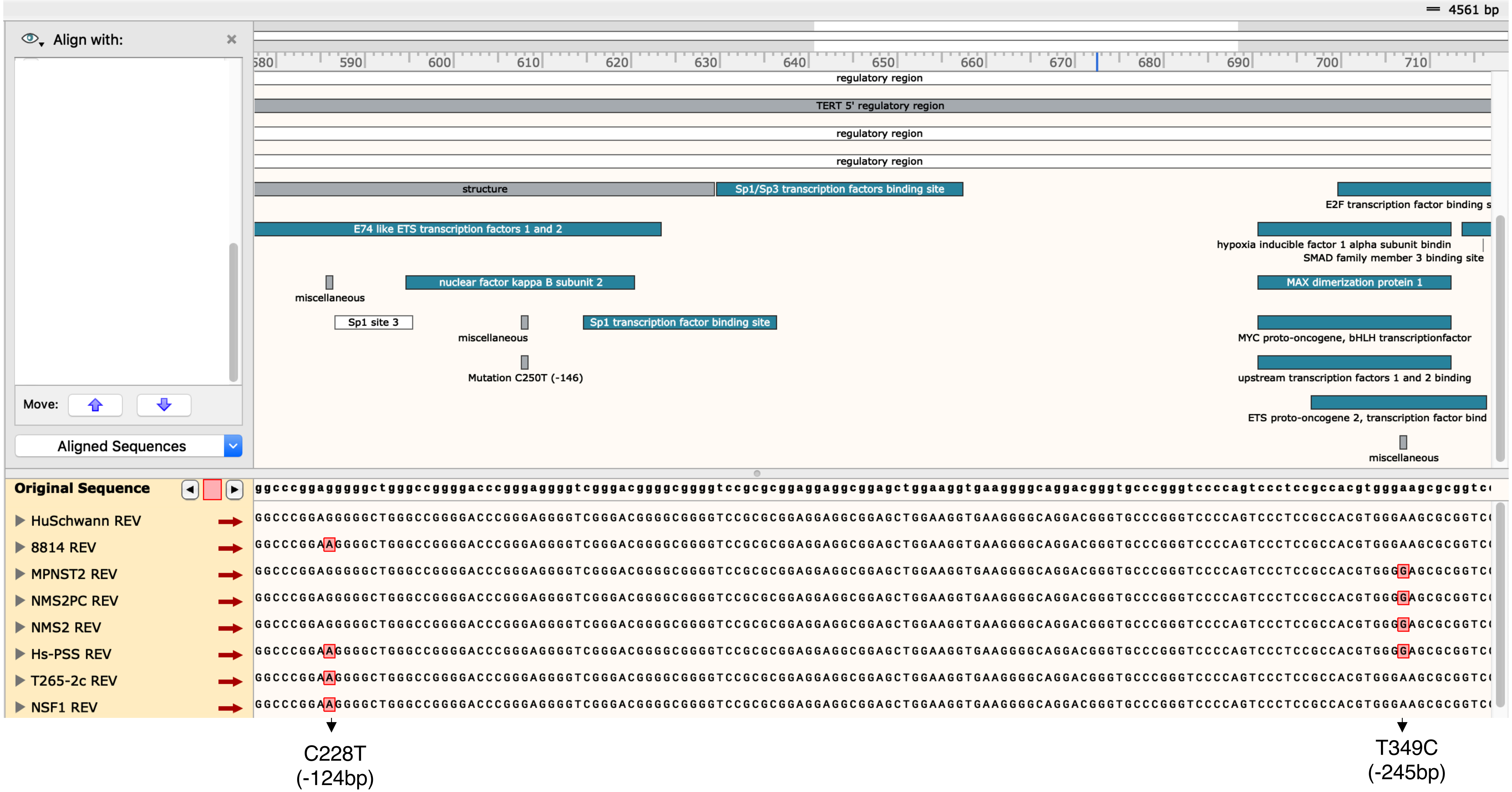

Supplemental Figure S9, Raw Images for western blots

Raf PD, NF1 immunoblot

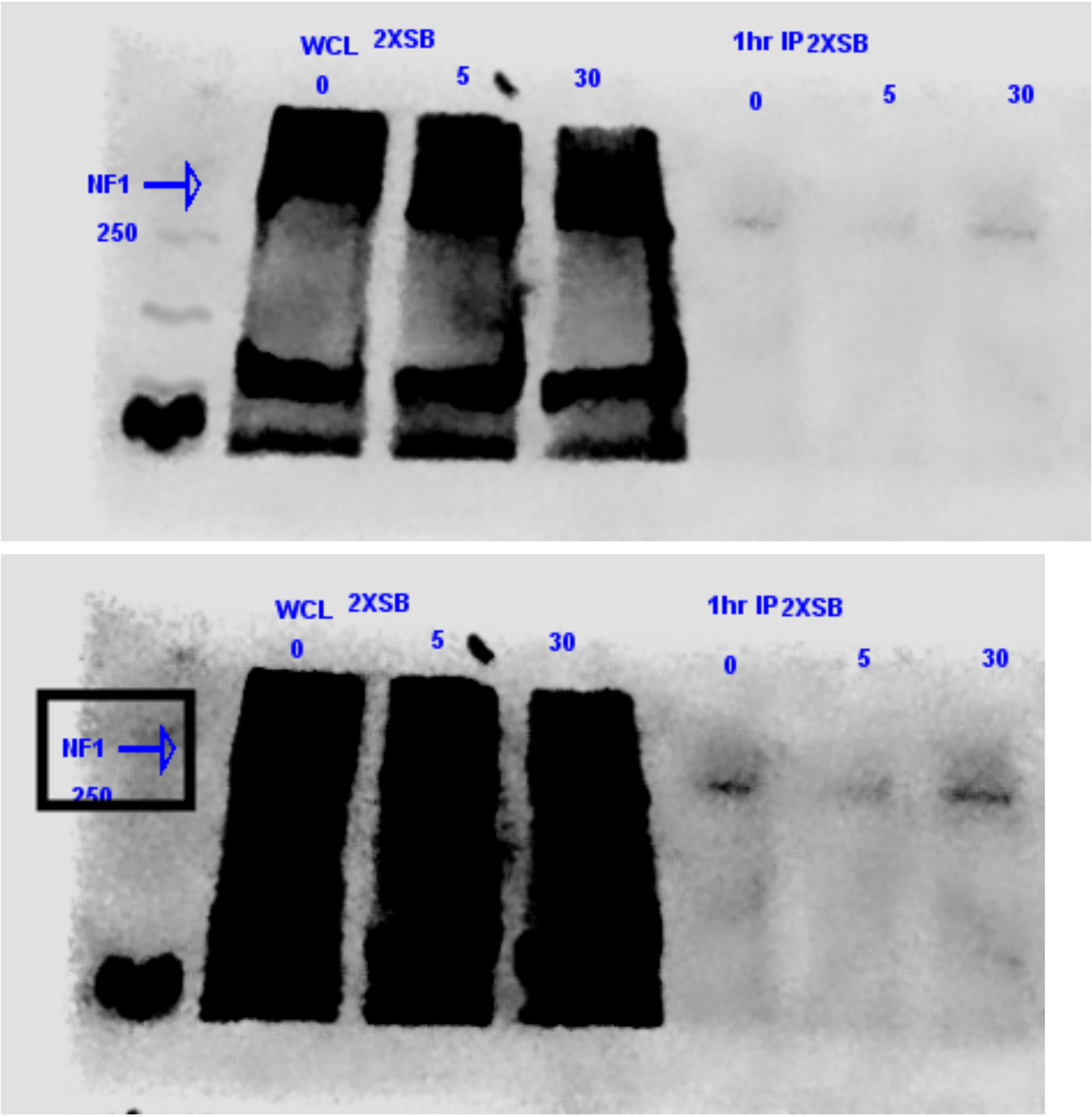

Light

Dark

Supplemental Figure S9, Raw Images for western blots

Raf PD, Ras immunoblot

Light

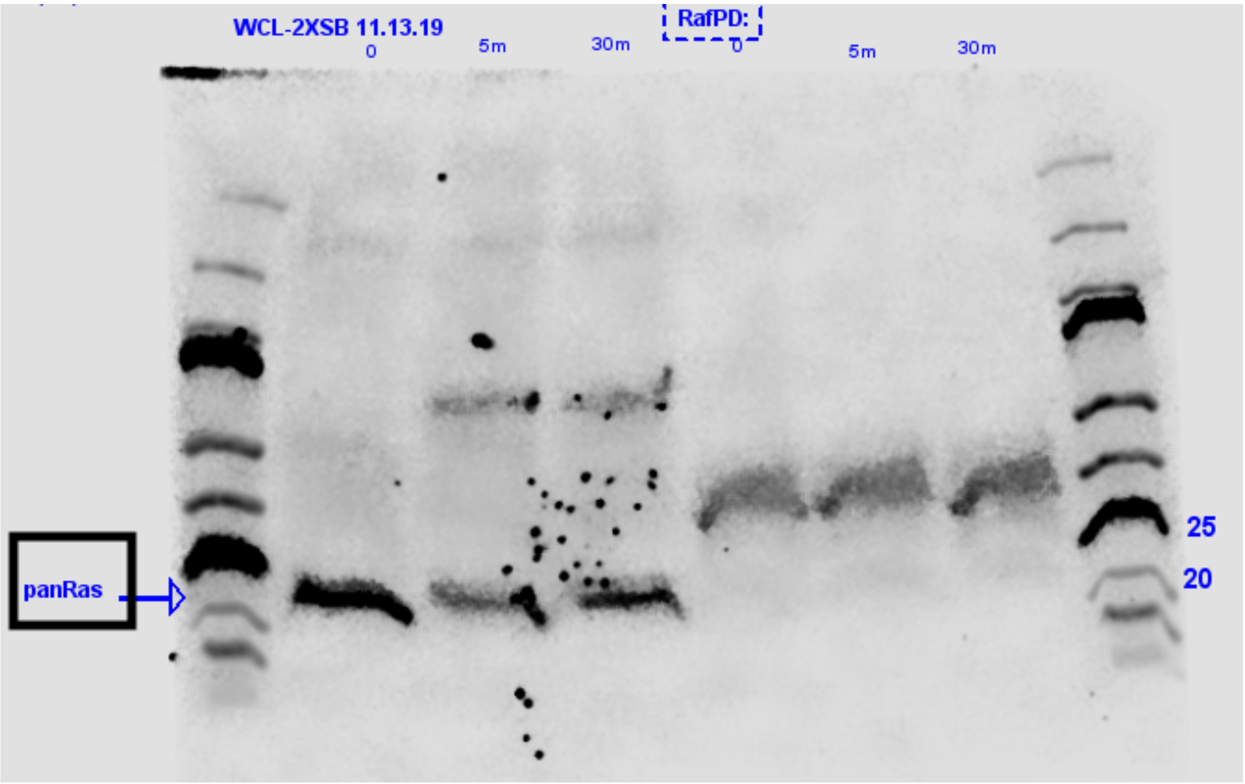

Dark

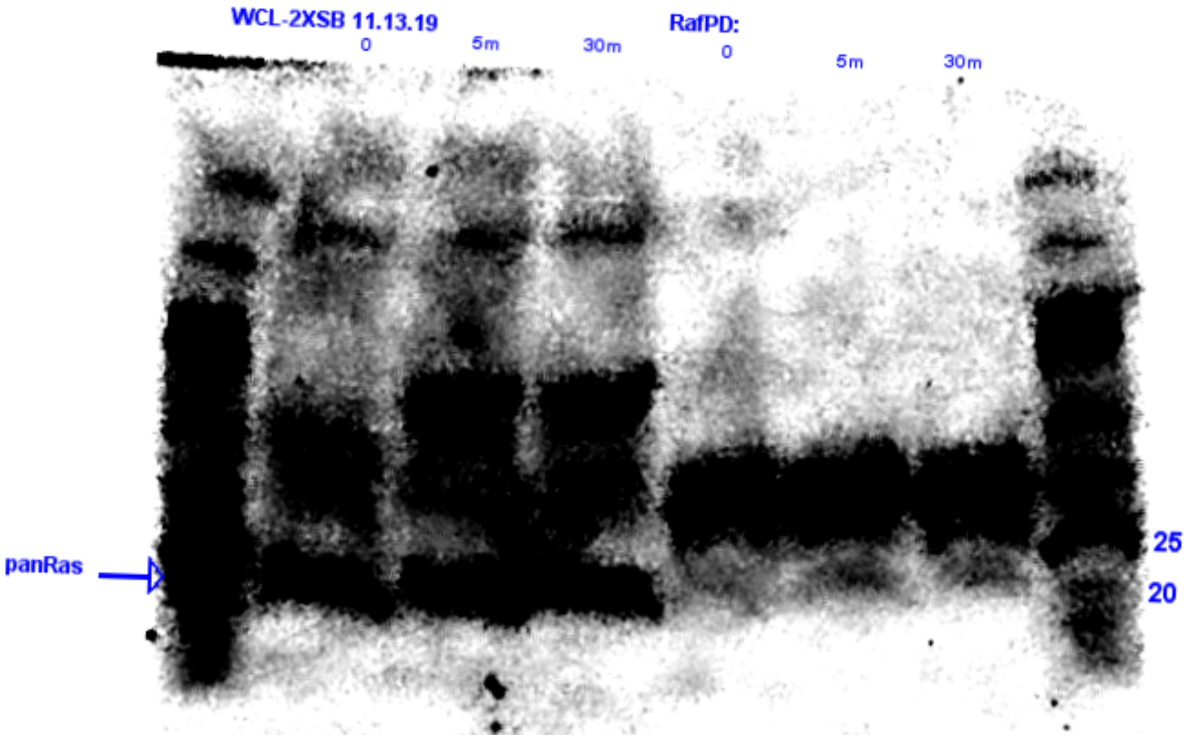

Supplemental Figure S9, Raw Images for western blots

Raf PD, CRAF, ERK immunoblot

dark

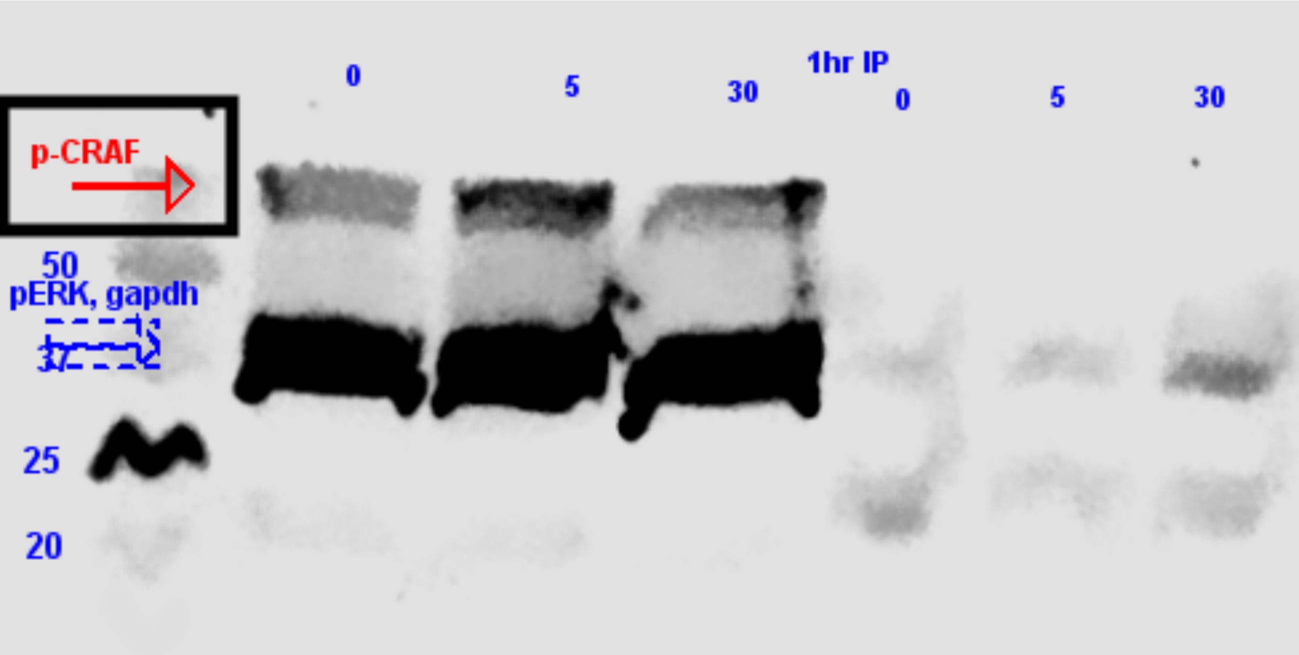

light

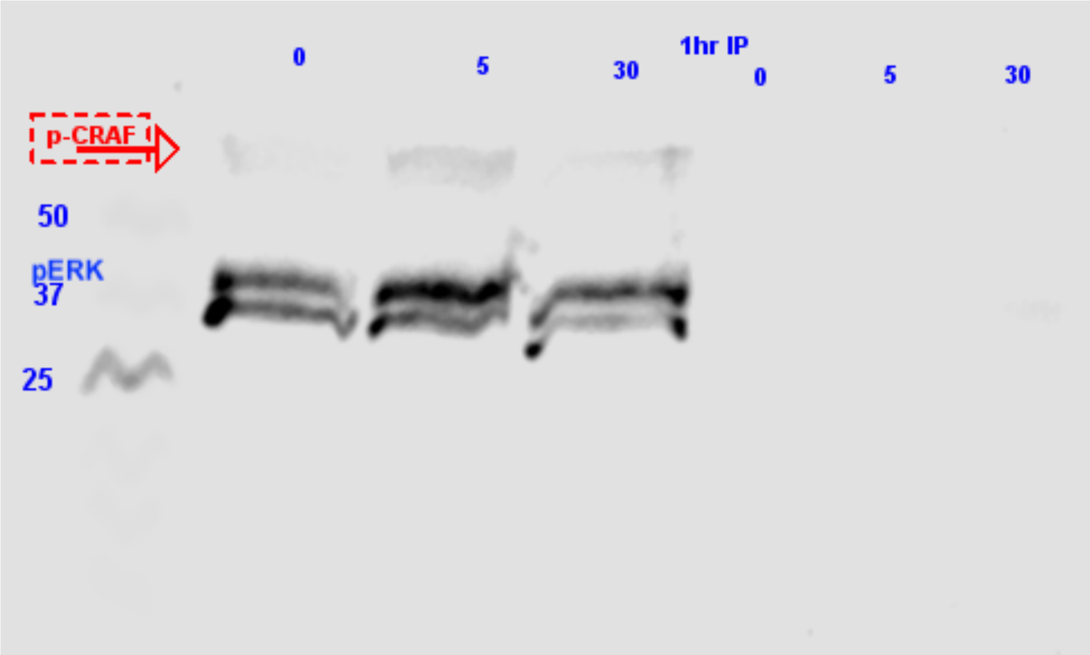

Supplemental Figure S9, Raw Images for western blots

Raf PD, gapdh immunoblot

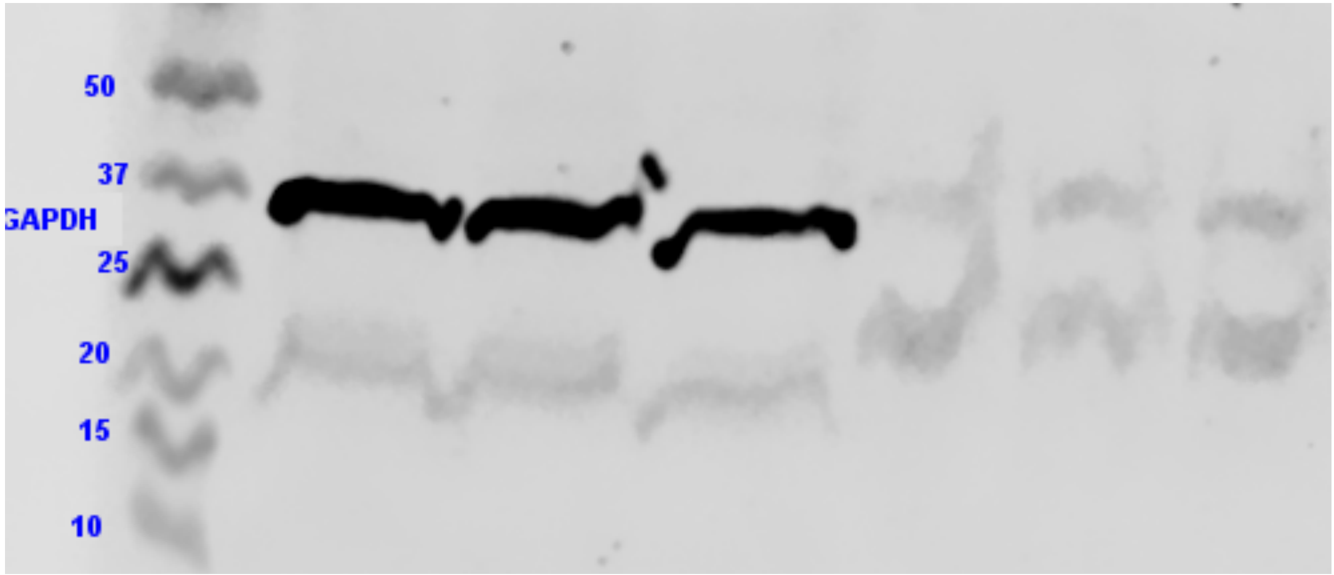

dark

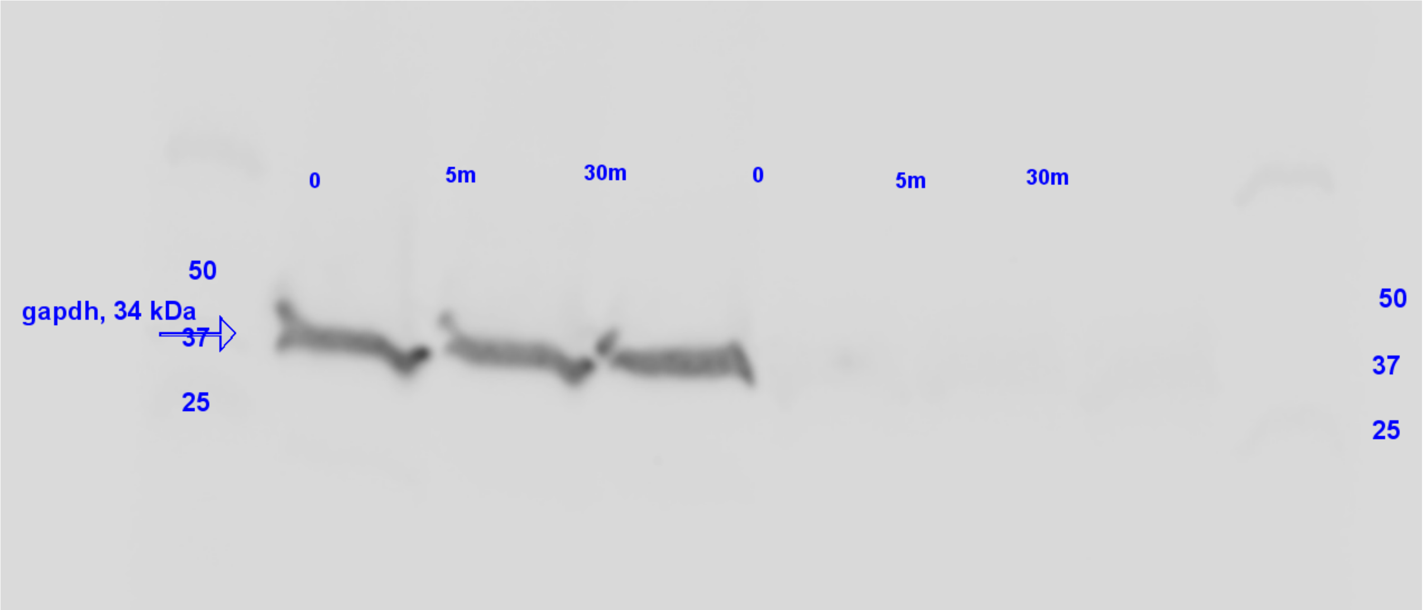

light

Supplemental Figure S9, Raw Images for western blots

Raf PD, total ERk immunoblot

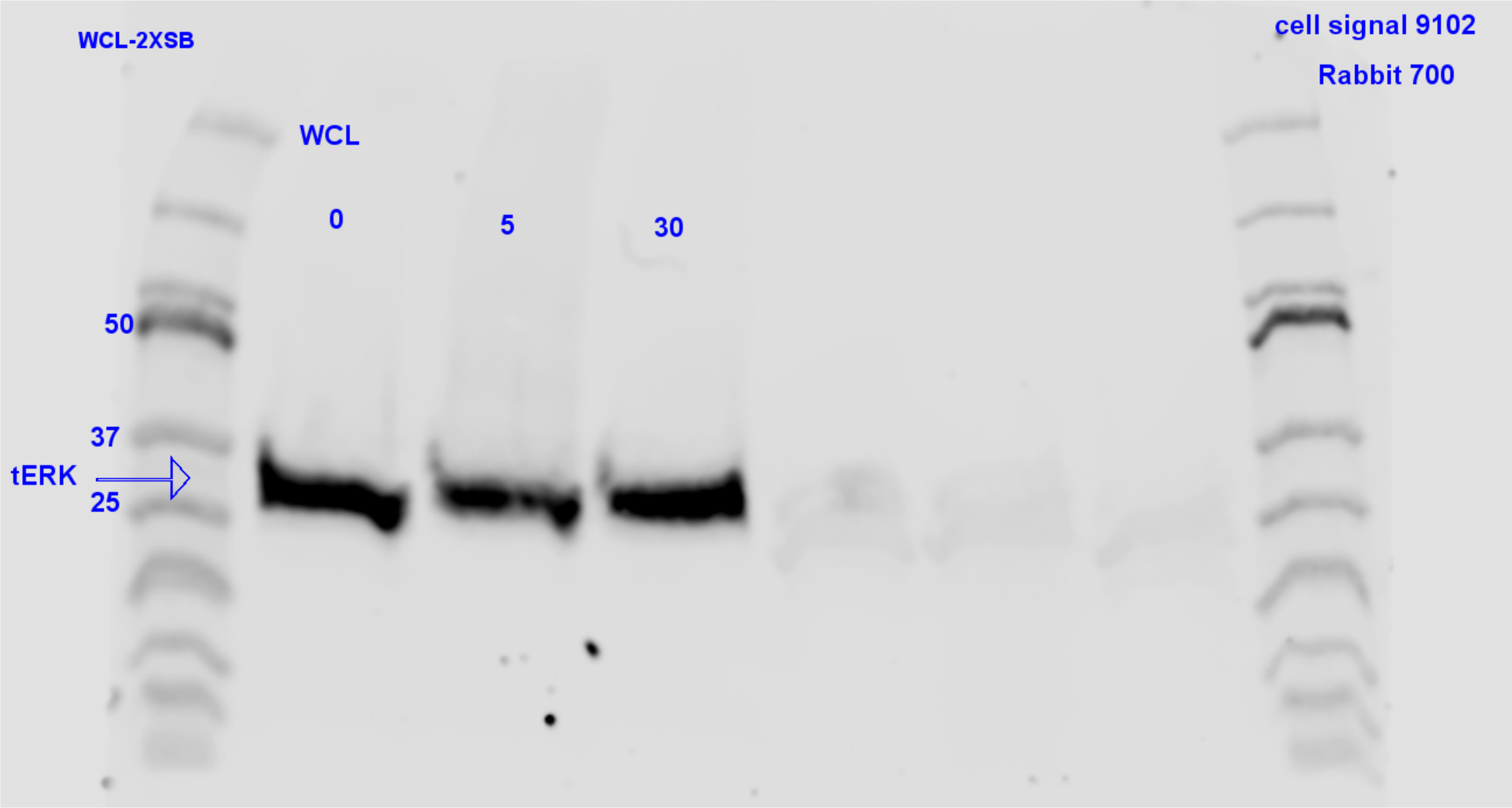

Supplement: Supplementary file 8 — Supplementary Figures. [file 41598_2021_85055_MOESM8_ESM.pdf]
